# Gamma-aminobutyric acid-producing lactobacilli positively affect metabolism and depressive-like behaviour in a mouse model of metabolic syndrome

**DOI:** 10.1038/s41598-019-51781-x

**Published:** 2019-11-08

**Authors:** E. Patterson, P. M. Ryan, N. Wiley, I. Carafa, E. Sherwin, G. Moloney, E. Franciosi, R. Mandal, D. S. Wishart, K. Tuohy, R. P. Ross, J. F. Cryan, T. G. Dinan, C. Stanton

**Affiliations:** 1 APC Microbiome Ireland, University College Cork, Cork, Ireland; 2 Teagasc Food Research Centre, Moorepark, Fermoy, Cork, Ireland; 3 Department of Food Quality and Nutrition, Research and Innovation Centre-Fondazione Edmund Mach, San Michele all’Adige, Trento, Italy; 4 Department of Biological Sciences, University of Alberta, Edmonton, Alberta Canada; 5 Department of Computing Science, University of Alberta, Edmonton, Alberta Canada; 6 National Institute for Nanotechnology, Edmonton, Alberta Canada; 7 Department of Anatomy and Neuroscience, University College Cork, Cork, Ireland; 8 Department of Psychiatry and Neurobehavioural Science, University College Cork, Cork, Ireland

**Keywords:** Microbiome, Metabolic syndrome, Type 2 diabetes

## Abstract

Metabolic and neuroactive metabolite production represents one of the mechanisms through which the gut microbiota can impact health. One such metabolite, gamma-aminobutyric acid (GABA), can modulate glucose homeostasis and alter behavioural patterns in the host. We previously demonstrated that oral administration of GABA-producing *Lactobacillus brevis* DPC6108 has the potential to increase levels of circulating insulin in healthy rats. Therefore, the objective of this study was to assess the efficacy of endogenous microbial GABA production in improving metabolic and behavioural outcomes in a mouse model of metabolic dysfunction. Diet-induced obese and metabolically dysfunctional mice received one of two GABA-producing strains, *L*. *brevis* DPC6108 or *L*. *brevis* DSM32386, daily for 12 weeks. After 8 and 10 weeks of intervention, the behavioural and metabolic profiles of the mice were respectively assessed. Intervention with both *L*. *brevis* strains attenuated several abnormalities associated with metabolic dysfunction, causing a reduction in the accumulation of mesenteric adipose tissue, increased insulin secretion following glucose challenge, improved plasma cholesterol clearance and reduced despair-like behaviour and basal corticosterone production during the forced swim test. Taken together, this exploratory dataset indicates that intervention with GABA-producing lactobacilli has the potential to improve metabolic and depressive- like behavioural abnormalities associated with metabolic syndrome in mice.

## Introduction

The obesity and metabolic dysfunction pandemic currently underway represents one of the most serious global health challenges of the 21^st^ century. According to the World Health Organization (WHO) in 2016, more than 1.9 billion adults were overweight, and of these, over 650 million were obese^1^. Attempts to curb this significant public health issue by creating efficacious primary and secondary prevention strategies have been largely unsuccessful due to the multifaceted nature of this disease. Failure in compliance with lifestyle interventions, coupled with novel environmental stressors, has rendered the global population susceptible to this pandemic. As a result, metabolic surgery is currently the most effective therapy for morbid obesity and metabolic disturbances^2^. This has driven the demand for researchers to investigate novel, non-invasive therapies for obesity and its associated sequelae. While significant inroads have been made in unravelling the biochemical and endocrinological underpinnings of obesity and its related comorbidities, derived translational therapeutic interventions have been sparse or are often limited by their side-effects^3^.

The composition, diversity and functionality of the gut microbiota are substantially discordant between healthy and obese subjects^4–6^. Thus, the gut microbiota has become a popular target for novel therapies directed towards obesity and diabetes, one which by-passes the need for significant lifestyle changes or surgery. Indeed, consumption of a high-fat (HF) diet and increased energy intake predisposes mice^7^ and humans^8^ to a low-grade inflammation, insulin resistance and considerable shifts in the microbiota. In turn, circulating bacterial particles such as lipopolysaccharide (LPS)^9,10^ are increased and trigger the host inflammatory immune response in tissues such as the liver and adipose tissue^7,11^. Furthermore, mice subcutaneously infused with LPS display increased susceptibility to weight gain and inflammation^11,12^. It is therefore plausible that changes in the composition of the gut microbiota may be intrinsically linked to the pathogenesis of obesity and related metabolic dysfunction.

Certain microbial therapeutics have proven efficacious in improving several physiological abnormalities associated with obesity and metabolic dysfunction in both preclinical^13–17^ and clinical studies^18,19^. As such, specific bacterial strains and their metabolites may be considered adjunct therapies for certain metabolic disorders. Microbial metabolites, such as short chain fatty acids and neuroactives, may be central to this effect. One such neurotransmitter produced by certain bacteria is gamma-aminobutyric acid (GABA), the primary inhibitory neurotransmitter in the central nervous system. Additionally, evidence suggests that this neurotransmitter may have a therapeutic role in treating type-1-diabetes (T1D) and long-standing type-2-diabetes (T2D) in which the pancreatic β-cells become fatigued. Activation of the GABA_A_ and GABA_B_ receptors expressed on β-cells has been shown to stimulate insulin secretion and induce β-cell proliferation^20–22^. Moreover, the GABA_A_ receptor is widely expressed on CD4+ T cells and several studies have indicated a putative immunomodulatory role of this neurotransmitter, suggesting an alternative mechanism through which it may impact upon systemic inflammation and the autoimmunity of T1D^23,24^.

Peripheral inflammation associated with obesity and metabolic dysfunction enhances the risk for neuropsychiatric and neurological disorders (for reviews see^25,26^). It has been shown that obese and T2D (*db/db*) mice display enhanced anxiety-like behaviour and impaired spatial recognition memory^27,28^, and we now understand that the gut microbiota plays a central role in gut-brain signalling which can impact brain physiology, function and behaviour^29^. In line with this, convincing pre-clinical evidence has demonstrated that enteric microbial GABA production can mediate physiological and behavioural effects via the vagus nerve^30^. However, little probing has been done into the behaviour-modifying attributes of GABA-secreting microbes in the context of obesity.

We previously isolated *L*. *brevis* DPC6108 from an infant faecal sample and *L*. *brevis* DSM32386 from a traditional Italian cheese, and demonstrated their ability to produce GABA *in vitro*^31,32^. We also explored the effects of intervention with *L*. *brevis* DPC6108 on the metabolic profile of two different rodent models; healthy and streptozotocin-induced T1D rats^33^. The aim of the present study was to assess whether microbial GABA production could attenuate the metabolic dysfunction observed in diet-induced obese mice. In addition, we aimed to determine whether microbial GABA production could attenuate impairments in the neuropsychiatric behavioural profile observed in these mice and associated with metabolic dysfunction.

## Results

### Survival of *L. brevis* DPC6108 and DSM32386 following gastrointestinal transit

Quantification of the number of colony forming units (CFUs) of administered rifampicin-resistant *L*. *brevis* DPC6108 and DSM32386 in the faeces of mice confirmed survival following gastrointestinal transit. Faecal recovery of *L*. *brevis* DPC6108 was ~2 × 10^9^ and *L*. *brevis* DSM32386 was ~1 × 10^9^ CFU/g faeces after two weeks and remained at similar numbers following four weeks of intervention. Colonies isolated from the plates were tested for GABA production to assess if gastrointestinal transit affected the ability of *L*. *brevis* DPC6108 and DSM32386 to produce GABA *in vitro*. Pooled fresh faecal samples collected from each cage of mice in DPC6108 and DSM32386 (4 cages per group) were analysed in duplicate following two and five weeks of intervention. At two and five weeks of intervention, bioconversion of MSG to GABA by DPC6108 was 68% and 83%, respectively (MRS containing 1% (w/v) MSG). At two and five weeks of intervention, bioconversion of MSG to GABA by DSM32386 was 66% and 78%, respectively. All colonies tested which were isolated from faecal samples had a similar percentage conversion of MSG to GABA. The average bioconversion of the wild-type DPC6108 and DSM32386 strains were 77% and 56% respectively. However, these averages are based on triplicates taken at one time-point only during the study and previous experimental analyses comparing the GABA-producing capabilities of these strain revealed no major differences between the wild-type strains. No significant differences were observed in the GABA-producing capabilities between the strains which had passed gastrointestinal transit compared to the wild-type strains.

### Intervention with GABA-producing microbes affected adipose deposition following HF diet

**Figure 1:**
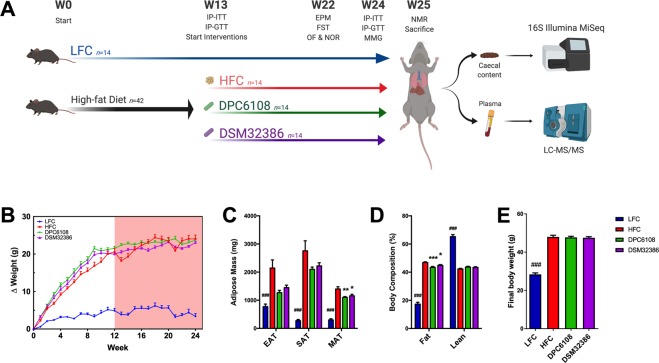
*L*. *brevis* reduced fat mass and distribution of adiposity after 12 weeks of intervention. (**A**) Schematic figure representing mouse trial design and timeline for procedures, with high-fat diet represented by the black arrow and treatments in a 12-week intervention represented by coloured arrows. (**B**) Bodyweight gain throughout the pre-feeding (white background) and intervention (red background) for n = 14 mice per group. (**C**) Adipose tissue mass and distribution across EAT, SAT and MAT and (**D**) Fat and lean body composition. (**E**) Final body weight after 24-week feeding (HFC; n = 13 and LFC; n = 10) and 12-week intervention with DPC6108 (n = 14) and DSM32386 (n = 14). Data are expressed as mean ± SEM. Bodyweight data were analysed using the mixed model ANOVA. Tukey’s post-hoc test was applied. All other data were analysed using the appropriate unpaired student t-test (HFC vs LFC) and one-way analysis of variance (ANOVA). ^###^*p* < 0.001 HFC vs LFC, *p < 0.05 treatment vs HFC, and **p < 0.01 treatment vs HFC. IP-GTT: intraperitoneal glucose tolerance test, IP-ITT: insulin tolerance test, EPM: elevated plus maze, FST: forced swim test, OF: open field, NOR: novel object recognition, MMG: mixed meal gavage, NMR: nuclear magnetic resonance, HFC: high fat control, DPC6108: *L*. *brevis* DPC6108, DSM32386: *L*. *brevis* DSM32386, LFC: low fat control, EAT: epididymal adipose tissue, SAT: subcutaenous adipose tissue and MAT: mesenteric adipose tissue. Figure 1A was created with BioRender.com.

Following 12 weeks of HF feeding, there were no significant differences in bodyweight gain over the intervention period between the HF groups (Fig. 1B). *L*. *brevis* DPC6108 (*p* < 0.01) and DSM32386 (*p* < 0.05) were associated with reduced quantity of mesenteric adipose tissue (MAT), compared with the high fat control (HFC; Fig. 1C). Both DPC6108 (*p* < 0.001) and DSM32386 (*p* < 0.05) reduced percentage body fat, compared with the HFC (Fig. 1D). There were no differences in final bodyweight observed between the groups consuming the HF diet (*F*_2,38_ = 0.108, *p* > 0.05; Fig. 1E). As expected, there was a significant difference in final bodyweight between the low fat control (LFC) and the HFC (*p* < 0.001; Fig. 1E).

### *L. brevis* DSM32386 improved HF diet-induced glucose intolerance and impaired insulin sensitivity

**Figure 2:**
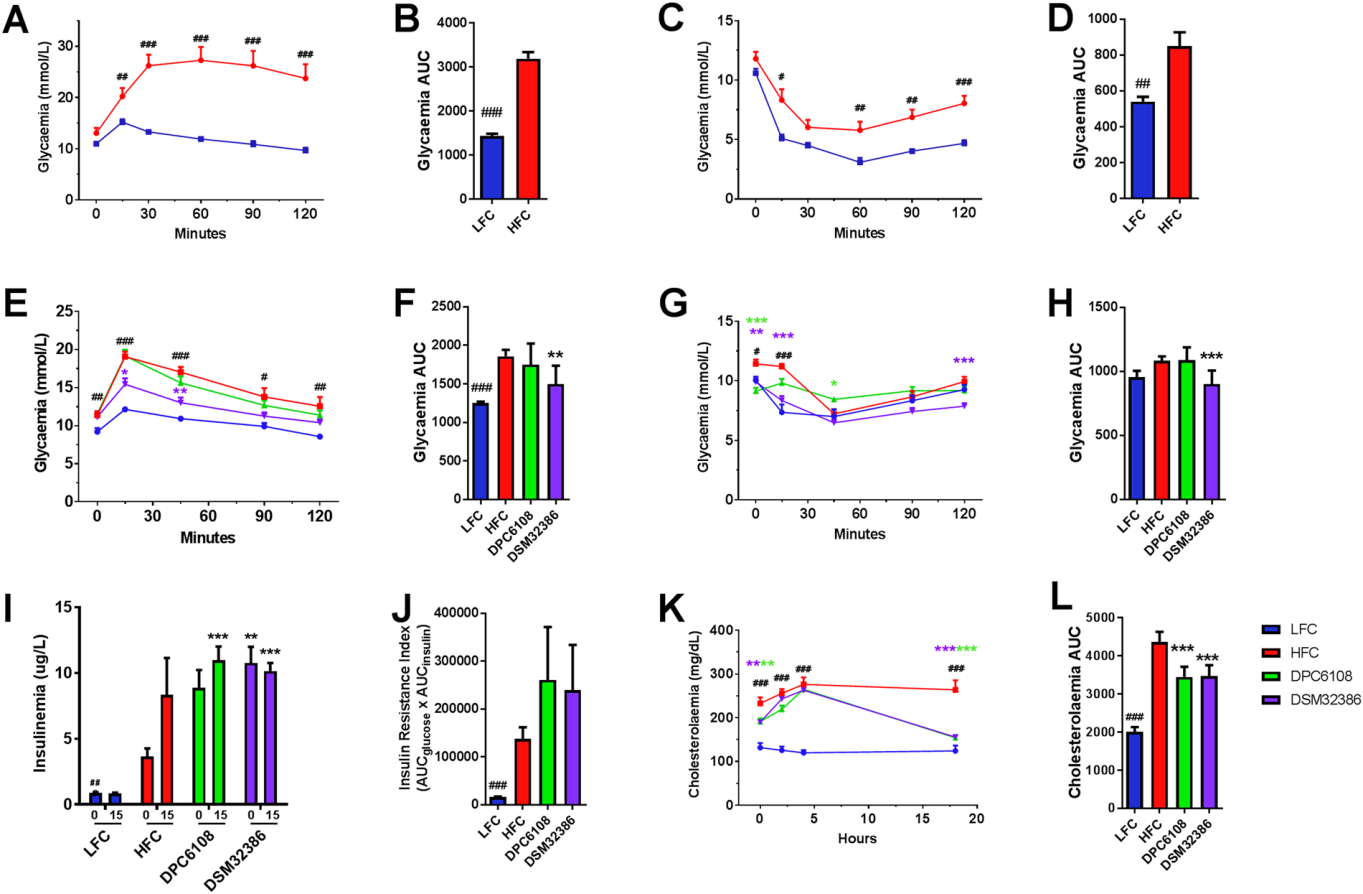
*L*. *brevis* DSM32386 improved glucose clearance in the IP-GTT while both strains improved glucose-dependent insulin secretion following glucose challenge in the IP-ITT. HFC (n = 7) and LFC (n = 7) mice were assessed for 1 g/Kg glucose (IP-GTT; **A**,**B**) and 0.75 IU/Kg insulin (IP-ITT; **C**,**D**) glycaemic responses following 12 weeks of feeding, prior to commencement of treatments at week 13. At week 24 of feeding and after 10 weeks of intervention, LFC (n = 7), HFC (n = 7), DPC6108 (n = 14), and DSM32386 (n = 14) mice were assessed for glycaemic responses to glucose (**E**,**F**) and insulin (**G**,**H**) challenges. Plasma insulin concentration as determined from samples collected at T = 0 and T = 15 during glucose challenge for LFC (n = 7), HFC (n = 5), DPC6108 (n = 14) and DSM32386 (n = 12) (**I**) and insulin resistance index determined by multiplying the area under the curve of both the blood glucose (0 to 120 min) and the plasma insulin (0 to 15 min) obtained following glucose challenge (**J**). At week 24 of feeding and after 10 weeks of intervention LFC (n = 7), HFC (n = 7), DPC6108 (n = 14), DSM32386 (n = 14) mice were assessed for cholesterol metabolism by oral gavage with a complete meal, Ensure Plus, Abbott Nutrition. Total plasma cholesterol was measured at T = 0, T = 2, T = 4 and T18 hr post-gavage (**K**). Total AUC for cholesterol metabolism was calculated from individual time points for all groups (**L**). Data are expressed as mean ± SEM. All data was analysed using the appropriate unpaired student t-test (HFC vs LFC) and one-way analysis of variance (ANOVA). ^#^*p* < 0.001 HFC vs LFC, ^##^*p* < 0.001 HFC vs LFC and ^###^*p* < 0.001 HFC vs LFC, *p < 0.05 treatment vs HFC, **p < 0.01 treatment vs HFC and ***p < 0.001 treatment vs HFC. AUC: area under curve, HFC: high fat control, DPC6108: *L*. *brevis* DPC6108, DSM32386: *L*. *brevis* DSM32386, LFC: low fat control.

After 12 weeks of HF feeding and prior to intervention with *L*. *brevis* (Fig. 1A), diet-induced hyperglycaemia and insulin insensitivity was confirmed in a subset of mice from the HFC, compared with a subset of mice from the LFC (Fig. 2A–D). After 12 weeks of HF feeding, the intraperitoneal glucose tolerance test (IP-GTT) revealed that HF-fed mice had higher blood glucose levels at 15 (t_6.804_ = 4.432 *p* < 0.01), 30 (t_5.520_ = 9.949, *p* < 0.001), 60 (t_6.580_ = 13.1, *p* < 0.001), 90 (t_10_ = 9.854, *p* < 0.001) and 120 (t_5.692_ = 7.326, *p* < 0.001); Fig. 2A) minutes, and a higher area under the curve (AUC) for glucose (t_6.226_ = 10.976, *p* < 0.001; Fig. 2B) compared with the LFC, indicating the impaired ability of HF-fed mice to respond to the glucose challenge. The intraperitoneal insulin tolerance test (IP-ITT) revealed that HF-fed mice were insensitive to exogenous insulin compared with the LFC (t_7.364_ = 3.783, *p* < 0.01; Fig. 2D). Therefore, 12 weeks of feeding with the HF diet was deemed sufficient to induce glucose intolerance (Fig. 2A,B) and insulin insensitivity (Fig. 2C,D), prior to microbial interventions.

Following an additional 10 weeks HF feeding in combination with *L*. *brevis* DPC6108 and *L*. *brevis* DSM32386, all mice underwent IP-GTT (Fig. 2E,F) and IP-ITT (Fig. 2G,H). After 22 weeks HF feeding, repeat IP-GTT revealed that mice in the HFC had higher fasting blood glucose levels at 0 (t_12_ = 3.077 *p* = 0.01), 15 (t_7.046_ = 10.358 *p* < 0.001), 45 (t_12_ = 8.101 *p* < 0.001), 90 (t_7.856_ = 3.173 *p* < 0.05) and 120 (t_12_ = 3.179 *p* < 0.01) minutes post-glucose load, compared with the LFC (Fig. 2E). The HFC also had a higher AUC for glucose (t_6.579_ = 7.137 *p* < 0.001), compared with the LFC (Fig. 2F). Somewhat in line with the results for the IP-GTT when comparing between HFC and LFC, the IP-ITT revealed a significant effect of HF diet on blood glucose levels at 0 (t_12_ = 2.930 *p* < 0.05) and 15 (t_12_ = 5.550 *p* < 0.001) minutes post-insulin challenge, compared with the LFC (Fig. 2G). There was only a marginally significant effect on the AUC for glucose between the HFC and LFC (t_12_ = 2.160 *p* = 0.052; Fig. 2H).

The microbial interventions had a significant effect overall on blood glucose levels at 15 (*F*_3,32_ = 7.390, *p* < 0.01) and 45 (*F*_3,32_ = 6.072, *p* < 0.01) minutes post-glucose load, compared with the HFC (Fig. 2E). DSM32386 mice displayed lower blood glucose levels at 15 (*p* < 0.05) and 45 (*p* < 0.01) minutes post-glucose load, compared with the HFC (Fig. 2E). There was a significant effect overall of the interventions on the AUC for the IP-GTT (*F*_2,32_ = 6.016, *p* < 0.01; Fig. 2F) and it was revealed that DSM32386 was the only intervention to significantly improve the ability to clear blood glucose following glucose challenge (*p* = 0.01; Fig. 2F). In line with this, the microbial interventions had a significant effect overall on blood glucose levels at 0 (*F*_3,32_ = 11.452, *p* < 0.001), 15 (*F*_3,32_ = 13.146, *p* < 0.001), 45 (*F*_3,32_ = 16.118, *p* < 0.001) and 120 (*F*_3,32_ = 8.776, *p* = 0.001) minutes post-insulin load during the IP-ITT, compared with the HFC (Fig. 2G). DSM32386 had lower blood glucose levels at 0 (*p* < 0.01), 15 (*p* < 0.001) and 120 (*p* = 0.001) minutes post-insulin load, compared with the HFC (Fig. 2G). DPC6108 had lower blood glucose levels at 0 (*p* < 0.001) and 45 (*p* < 0.05) minutes post-insulin load, compared with the HFC (Fig. 2G). Overall, intervention with both *L*. *brevis* strains had a significant effect on total AUC during the IP-ITT (*F*_2,32_ = 14.408, *p* < 0.001; Fig. 2H) and DSM32386 improved insulin sensitivity (*p* = 0.001; Fig. 2H).

Plasma was collected during T0 and T15 of the IP-GTT to measure insulin and, subsequently, insulin resistance index (Fig. 2I,J). At 0 minutes, before glucose load, there was an overall effect of *L*. *brevis* on plasma insulin (*F*_2,30_ = 5.46, *p* = 0.01; Fig. 2I). Intervention with DSM32386 increased resting plasma insulin at 0 minutes (*p* < 0.01), compared with the HFC (Fig. 2I). Insulin production 15 minutes post glucose load was higher in both microbial interventions (DPC6108; *p* < 0.001 and DSM32386 (*p* < 0.001), compared with the HFC (Fig. 2I). No differences in the insulin resistance index were observed following microbial intervention, compared with the HFC (Fig. 2J).

### GABA-producing microbes improved post-prandial cholesterol metabolism

Assessment of the plasma at T0, prior to the mixed meal gavage (MMG) challenge indicated that microbial intervention had a significant impact on fasted plasma cholesterol levels (F_2,32_ = 6.931, *p* < 0.01), compared with the HFC (Fig. 2K). Post-hoc analysis revealed DPC6108 (*p* < 0.01) and DSM32386 (*p* < 0.01) reduced cholesterol levels at T0, compared with the HFC (Fig. 2K). No significant impact of intervention on plasma cholesterol was identified at two or four hours post-MMG; however, at 18 hours DPC6108 (*p* < 0.001) and DSM32386 (*p* < 0.001) both displayed reduced cholesterol levels, compared with the HFC (Fig. 2K). There was a significant effect overall of microbial intervention on total AUC for plasma cholesterol throughout the MMG (F_2,13.573_ = 14.547, *p* < 0.001, Fig. 2L). DPC6108 (*p* < 0.001) and DSM32386 (*p* < 0.001) significantly reduced plasma cholesterol levels, compared with the HFC (Fig. 2L).

### GABA-producing microbes increased luminal GABA levels in the small intestinal content

**Figure 3:**
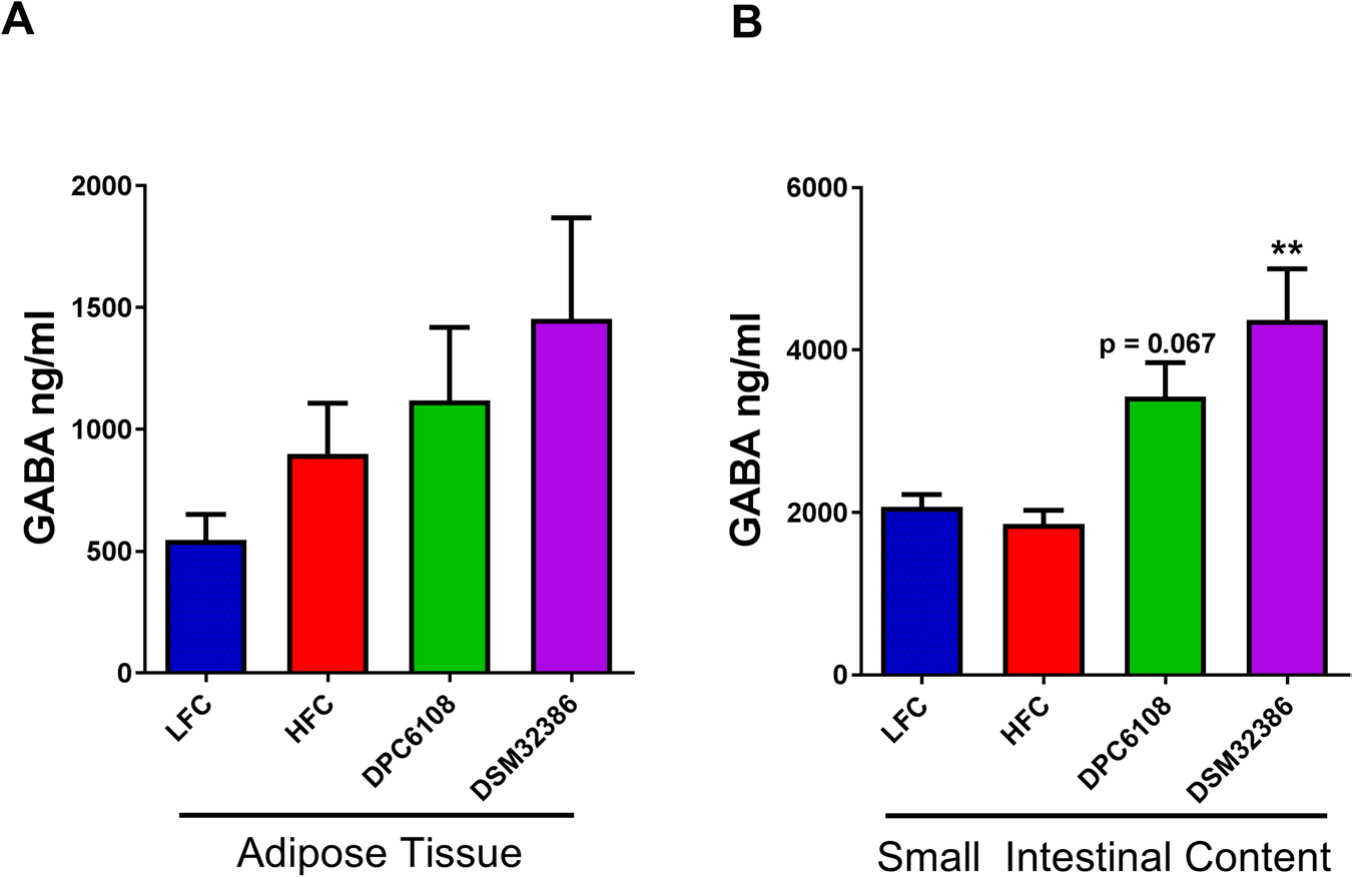
*L*. *brevis* increased GABA levels in small intestinal content. GABA levels were determined from tissue samples collected at the end of the 12-week intervention from LFC (n = 8), HFC (n = 12), DPC6108 (n = 14), DSM32386 (n = 11) from epididymal adipose tissue (**A**) and from LFC (n = 10), HFC (n = 12), DPC6108 (n = 14), DSM32386 (n = 14) in total small intestinal content (**B**). Data are expressed as mean ± SEM. All data was analysed using the appropriate unpaired student t-test (HFC vs LFC) and one-way analysis of variance (ANOVA). ^###^*p* < 0.001 HFC vs LFC, **p < 0.01 treatment vs HFC. AUC: area under curve, GABA: gamma-aminobutyric acid, HFC: high fat control, DPC6108: *L*. *brevis* DPC6108, DSM32386: *L*. *brevis* DSM32386, LFC: low fat control.

Diet had no impact on either adipose tissue (t_15.738_ = 1.522) or small intestinal content (t_20_ = −0.898) of GABA, between the HFC and LFC groups (Fig. 3A,B). This was unsurprising as both diets were matched for amino acid profiles. *L*. *brevis* did have a significant impact on small intestinal content of GABA (F_2,20.551_ = 6.906, *p* < 0.001), compared with the HFC (Fig. 3B). While intervention with DPC6108 had a tendency to increase luminal contents of GABA (*p* = 0.067), DSM32386 significantly increased GABA (*p* < 0.01) in the small intestine, compared with the HFC (Fig. 3B).

### GABA-producing microbes had little effect on host plasma metabolome

**Figure 4:**
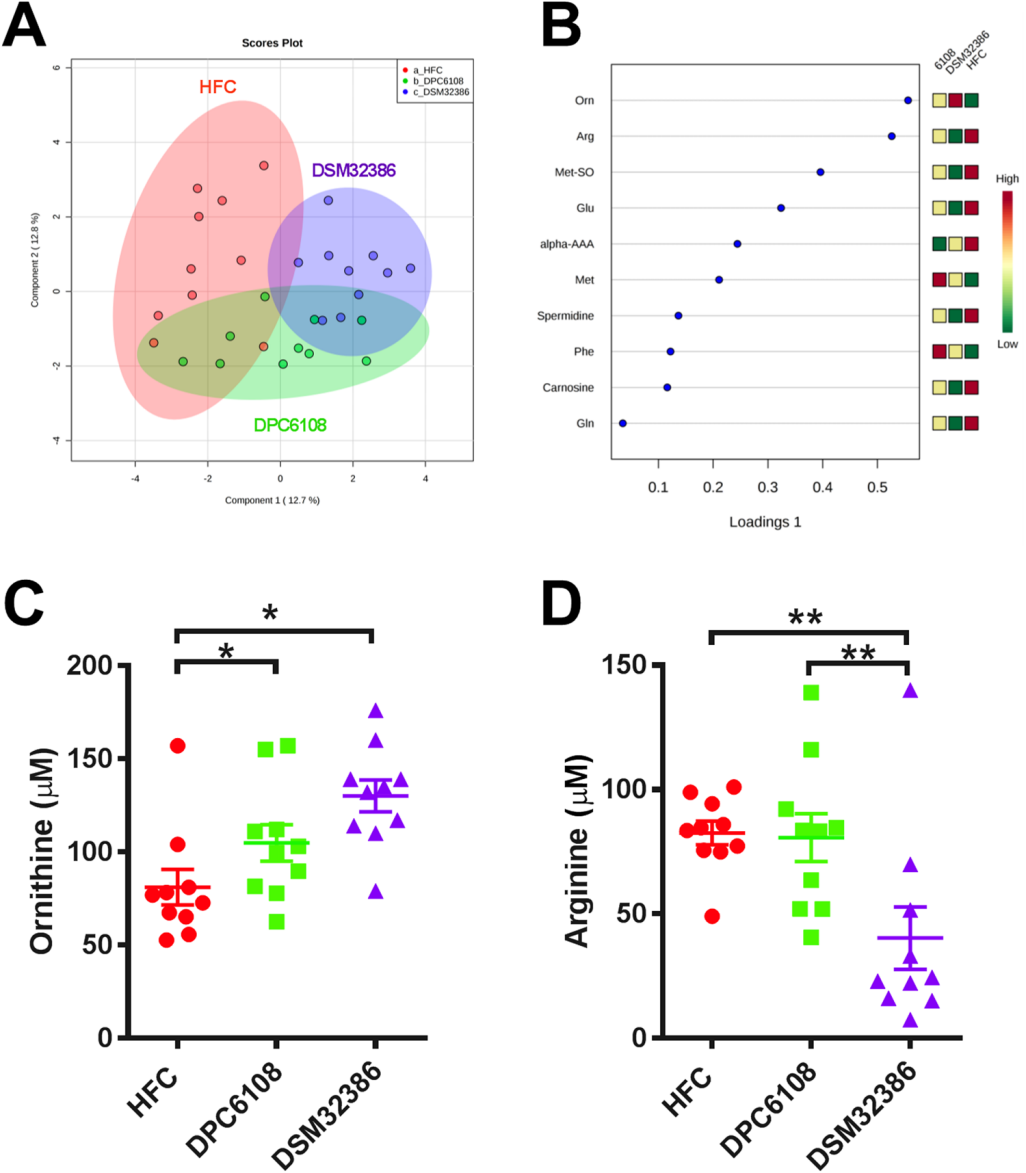
*L*. *brevis* altered plasma ornithine and arginine. (**A**) Sparse partial least square discriminant analysis (sPLS-DA) plot displays HFC (n = 10; red), DPC6108 (n = 10; green) and DSM32386 (n = 10; purple) samples. (**B**) Plot metabolite loadings graph displaying key metabolite differences between groups. Quantitative data is displayed for the metabolites which were significantly altered by DPC6108 or DSM32386; this included the biogenic amine ornithine (**C**) and amino acid arginine (**D**). Orn:Ornithine, Arg:Arginine, Met-SO:Methioninesulfoxide, Glu:Glutamate, alpha AAA:alpha Aminoadipic Acid, Met:Methionine, Phe:Phenylalanine, Gln:Glutamine, HFC: high fat control, DPC6108: *L*. *brevis* DPC6108, DSM32386: *L*. *brevis* DSM32386. *q < 0.05 treatment vs HFC, **q < 0.01 treatment vs HFC. Plots depict individual replicates with mean and SEM.

Targeted analysis of 140 metabolites from the plasma metabolome taken from fasted mice unveiled modest metabolic alterations as a result of microbial intervention. Figure 4A depicts a sparse partial least square discriminant analysis (sPLS-DA) plot, with the metabolites most important to the model displayed in the loadings plot (Fig. 4B). DPC6108 had little impact on the plasma metabolome, solely increasing the biogenic amine ornithine when compared with the HFC (*q* < 0.05; Fig. 4C). DSM32386 also increased ornithine compared with the HFC (*q* < 0.05; Fig. 4C), while decreasing arginine compared to both the HFC and DPC6108 (*q* < 0.01; Fig. 4D).

### Diet and GABA-producing microbes had a significant impact on gut microbial composition and diversity

A total of 51 faecal samples were subjected to 16S metataxanomic analysis. Illumina sequencing generated a total of 2,560,853 high quality sequences. After rarefying the sequencing depth, Chao 1 diversity index and observed operational taxonomy units (OTU) were calculated (Supplementary Table S2). Chao 1 index showed a higher diversity profile in the LFC (mean value = 4,397.0, *p* < 0.05) and DPC6108 (mean value = 4,138.5, *p* < 0.05), compared with the HFC. Moreover, DPC6108 showed higher diversity, compared with the HFC. The data show that there was a significant difference in the richness, with reduced numbers of OTUs in all HF diet-fed groups, compared with the LFC; although the number of OTUs was increased in DPC6108, compared with the HFC. To assess beta-diversity, Bray-Curtis distance matrix was calculated and differences were identified between LFC (*p* < 0.001) and DSM32386 (*p* < 0.05), compared with the HFC (Supplementary Table S2).

**Figure 5:**
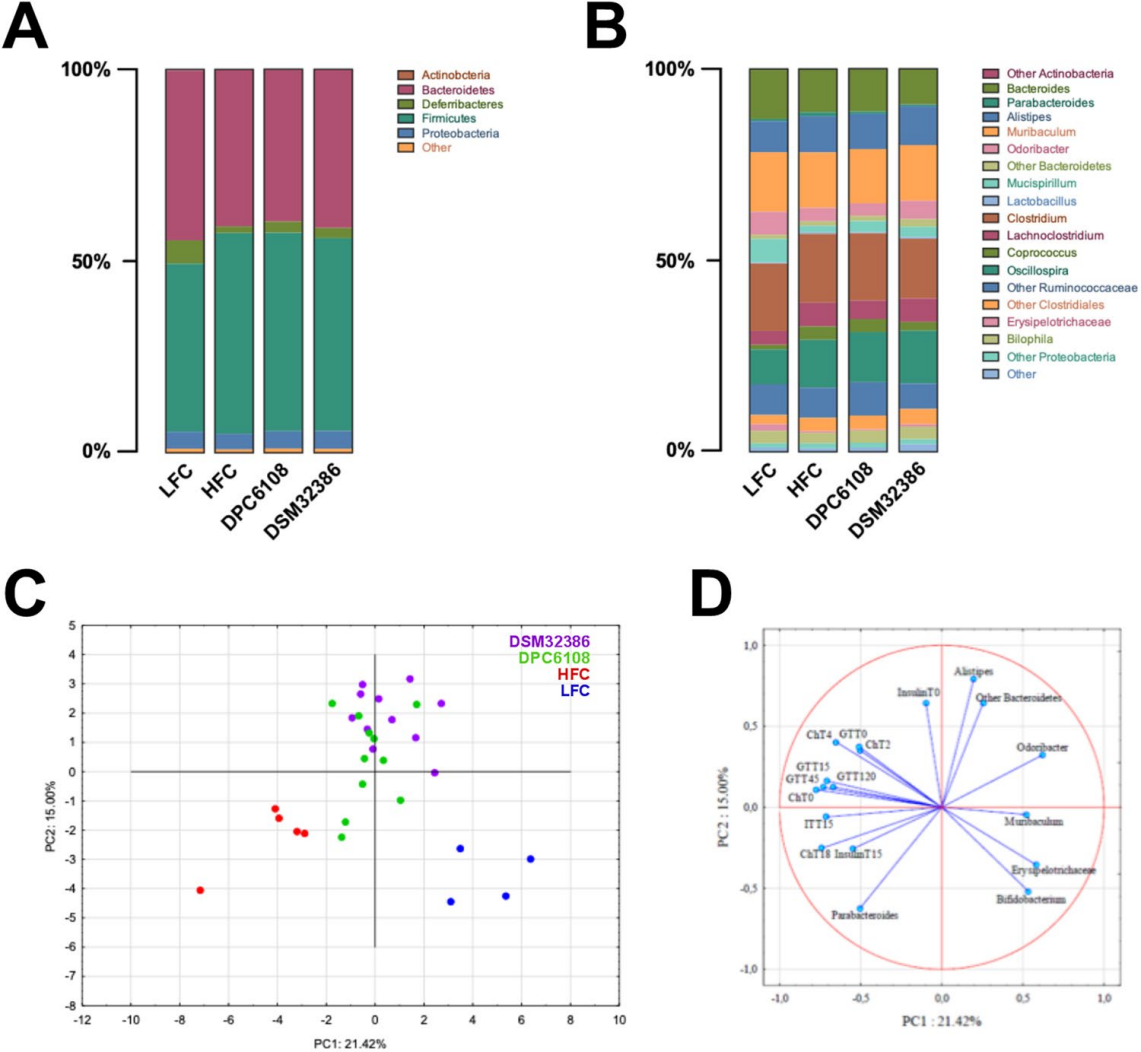
Both diet and *L*. *brevis* had a significant impact on gut microbiota composition. Relative abundance of microbial taxa at (**A**) phylum and (**B**) genus level. Phyla and genera representing <0.1% abundance in any sample were considered as Other. The PCA plot based on OTU relative abundances of the bacterial community and the metabolic parameters measured in mice (**C**). The first two principal coordinates (PC1 and PC2) are plotted for each sample (**D**), with the variables related to the variance between groups. PCA: Principal Component Analysis, OTU; Operational Taxonomy Unit.

At phylum level, all groups demonstrated comparable microbial compositions (Fig. 5A). In LFC, Firmicutes (43.8%) and Bacteroidetes (44.3%) were dominant (ratio Firmicutes/Bacteroides = 0.98). The phylum Deferribacteres (6.1%) was significantly higher in LFC, compared with all HF diet-fed groups. A reduction of Deferribacteres (1.7%, *p* < 0.001) and increase of Firmicutes (52.3%, *p* < 0.05) was observed in the HFC, compared with the LFC. HFC, DSM32386 and DPC6108 mice had similar relative abundance of Firmicutes and Bacteroidetes (ratio Firmicutes/Bacteroides HFC = 1.28, DSM32386 = 1.22, DPC6108 = 1.30), while the relative abundance of Deferribacteres was higher in DSM32386 (2.7%, *p* = 0.09) and DPC6108 (2.9%, *p* = 0.08), compared with the HFC (1.7%).

At the genus level (Fig. 5B), *Akkermansia* was higher in the LFC (0.02%), compared with the HFC (0.01%; *p* < 0.0001), but absent in both DSM32386 and DPC6108. A higher abundance of *Odoribacter* (6.02%) and a lower abundance of *Lachnoclostridium* (3.57%) were detected in the LFC, compared with the HFC (*Odoribacter p* < 0.001, *Lachnoclostridium p* = 0.01), DSM32386 (*Odoribacter p* < 0.05, *Lachnoclostridium p* = 0.05) and DPC6108 (*Odoribacter p* < 0.001). *Oscillospira* and *Clostridiales_other* were higher in the HFC (*Oscillospira p* = 0.01, *Clostridiales_other p* < 0.01), DSM32386 (*Oscillospira p* < 0.01, *Clostridiales_other p* = 0.001) and DPC6108 (*Oscillospira p* < 0.05, *Clostridiales_other p* < 0.001), compared with the LFC (1.17%, 9.07%, 2.43%, respectively). All sequences associated to the genus *Lactobacillus* were blasted manually on the NCBI Basic Local Alignment Search Tool and the species *L*. *brevis* was detected only in DSM32386 and DPC6108 groups.

### *Bifidobacterium*, *Erysipelotrichaceae*, *Muribaculum* and *Odoribacter* were related to reduced glycaemia and cholesterolaemia

The principal component analysis (PCA) plot (Fig. 5C) and correlation matrix (Fig. 5D) were generated based on the relative microbial composition and metabolic parameters. Correlations between microbiota and biochemical analyses revealed that the increase of certain microbial taxa corresponded to the improvement or deterioration of glycaemia (after IP-GTT or IP-ITT), insulin or cholesterol levels (Supplementary Table S3). Indeed, diet appeared to be the primary determinant of these microbiota-biochemistry associations, as both are known to be substantially impacted by such factors. The PCA scores clearly separated the LFC from the HF groups, with DSM32386 and DPC6108 clustering together away from the HFC (Fig. 5C). PCA1 and PCA2 explained the 21.42% and 15.0% of variance between groups, respectively. The variance associated to the LFC was related to *Erysipelotrichaceae*, *Muribaculum* and *Bifidobacterium* on PCA1 (|loadings| ≥ 0.52), and *Bifidobacterium* on PCA2 (|loadings| ≥ 0.52). The variance associated to DSM32386 and DPC6108 was related to *Odoribacter*, cholesterol (T0, T2 and T4), glycaemia under IP-GGT (at T0, T15, T45, T120) on PCA1 (|loadings| ≥ 0.50) and *Alistipes*, other *Bacteroidetes*, and resting insulin on PCA2 (|loadings| ≥ 0.64), apart from four samples belonging to DPC6108 (Fig. 5D).

### GABA-producing microbes improved depression-like behaviour and basal corticosterone levels during the forced swim test (FST)

**Figure 6:**
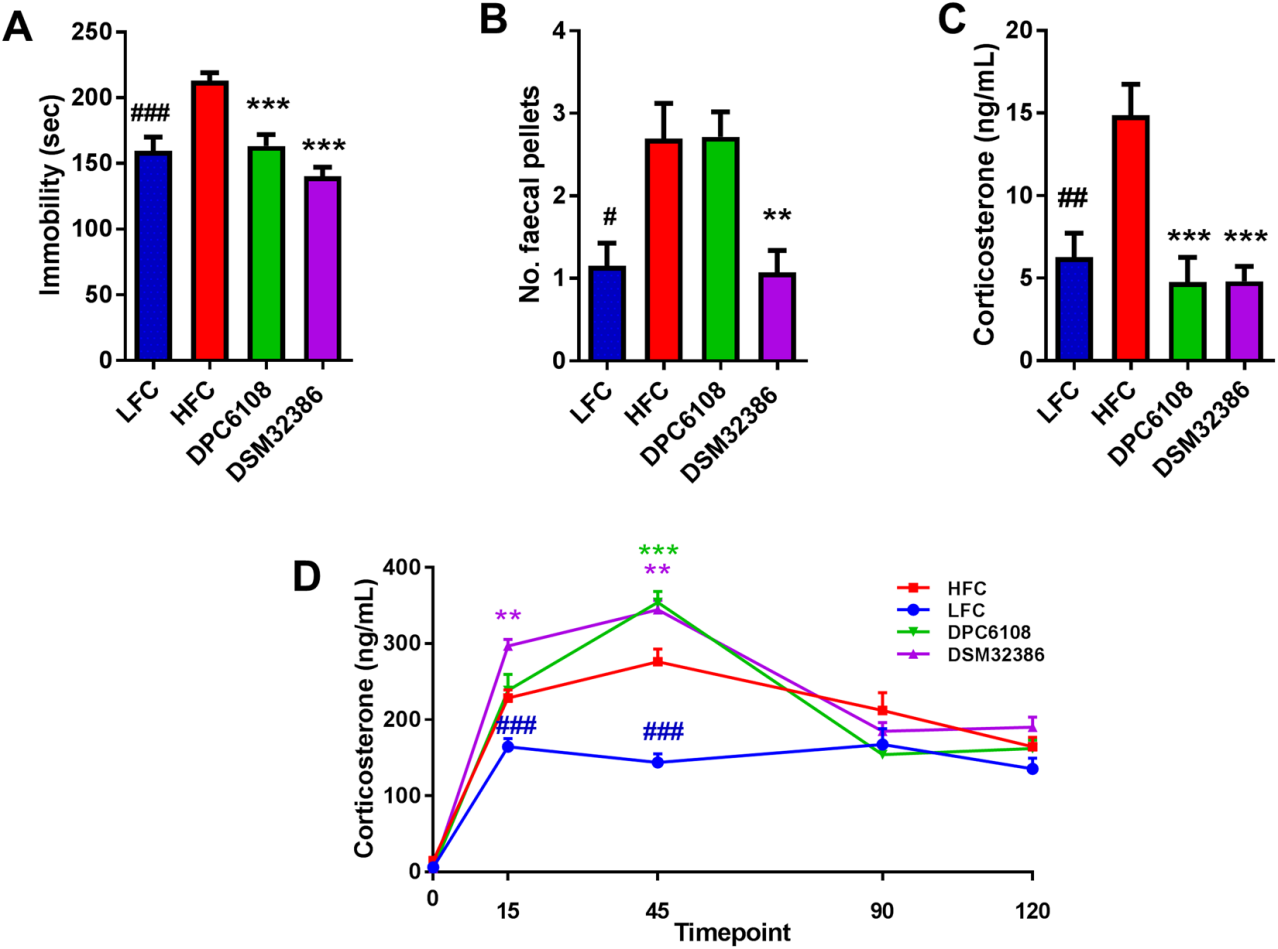
*L*. *brevis* improved symptoms of depression-like behaviour in the FST and resting corticosterone levels. The effect of HF-diet feeding and *L*. *brevis* intervention on depression-like behaviour and the HPA axis response to stress was assessed after 21 weeks of feeding and after 8 weeks of intervention. Despair behaviour was assessed thorough immobility time (**A**) by mice throughout the test and anxiety-like behaviour was assessed through the number of faecal pellets produced during the test (**B**). Plasma isolated at T = 0, just prior to the FST was used to measure corticosterone production (**C**) and subsequent plasma samples collected at T = 15, T = 45, T = 90 and T = 120 post-FST were used to calculate corticosterone as a measure of the HPA axis response to stress (**D**). Samples were collected from mice in LFC (n = 13), HFC (n = 12), DPC6108 (n = 11), DSM32386 (n = 14). All data was analysed using the appropriate unpaired student t-test (HFC vs LFC) and one-way analysis of variance (ANOVA). ^#^*p* < 0.05 HFC vs LFC, ^##^*p* < 0.01 HFC vs LFC and ^###^*p* < 0.001 HFC vs LFC, **p < 0.01 treatment vs HFC and ***p < 0.001 treatment vs HFC. FST: forced swim test, HF: high fat, HFC: high fat control, HPA: hypothalamic pituitary adrenal, DPC6108: *L*. *brevis* DPC6108, DSM32386: *L*. *brevis* DSM32386, LFC: low fat control.

Diet and bodyweight impacted immobility time during the FST. Mice in the HFC group spent more time immobile, compared with the LFC (t_21_ = 4.245, *p* < 0.001; Fig. 6A). While it was hypothesised that obesity itself impaired mobility in the HFC, data from the novel object recognition (NOR)/open field test would suggest that bodyweight had no significant effect on mobility (Fig. S2D). Therefore, depression-like behaviour was induced through consumption of the HF diet. *L*. *brevis* had an overall impact on immobility time during the FST (F_2,36_ = 19.474, *p* < 0.001), compared with the HFC (Fig. 6A). Post-hoc analysis revealed that DPC6108 (*p* < 0.001) and DSM32386 (*p* < 0.001) reduced the duration of time spent immobile during the FST, compared with the HFC (Fig. 6A). In addition, mice in the HFC group produced more faecal pellets during the FST (t_24_ = 3.021, *p* < 0.05), compared with the LFC, indicative of enhanced anxiety during this test (Fig. 6B). Finally, DSM32386 produced significantly fewer faecal pellets during the FST (*p* < 0.01), compared with the HFC (Fig. 6B).

Plasma was isolated from individual mice at 0 minutes pre-FST, then at 15, 45, 90 and 120 minutes post-FST to assess the impact of diet and microbial intervention on the hypothalamic-pituitary-adrenal (HPA) axis response to stress. Basal corticosterone levels were increased in the HFC, compared with the LFC (t_23_ = 3.619, *p* < 0.01; Fig. 6C). Interestingly, *L*. *brevis* restored basal corticosterone to levels comparable with the LFC and prevented the apparent HF dietary stimulation of the HPA axis and the stress response (F_2,36_ = 16.110, *p* < 0.001; Fig. 6C). DPC6108 (*p* < 0.001) and DSM32386 (*p* < 0.001) reduced basal corticosterone levels, compared with the HFC (Fig. 6C).

Following the FST, the HFC had higher levels of corticosterone at 15 (t_21_ = 4.104, *p* < 0.001) and 45 (t_22_ = 6.692, *p* < 0.001) minutes, compared with the LFC (Fig. 6D). In addition, DPC6108 at 45 (*p* = 0.001), and DSM32386 at 15 (*p* < 0.01) and 45 (*p* < 0.01) minutes post-FST displayed higher corticosterone levels, compared with the HFC (Fig. 6D).

### Diet and bodyweight affected mobility, while GABA-producing microbes had no effect on anxious behaviour

Diet and bodyweight had a significant impact on mobility, represented by total distance travelled during the aversive open-field (OF) test (Fig. S1A). Mice in the HFC group travelled significantly less in the open arena, compared with the LFC (t_24_ = 3.678, *p* < 0.01; Supplementary Fig. S1A). *L*. *brevis* had no impact on the total distance travelled, compared with the HFC (Supplementary Fig. S1A). There was no effect of diet (HFC vs LFC) on duration of time spent in inner (Supplementary Fig. S1B) or outer (Supplementary Fig. S1C) zones of the aversive OF. In addition, *L*. *brevis* had no effect on the duration of time spent in inner (Supplementary Fig. S1B) and outer zones (Supplementary Fig. S1C) of the OF. There was no effect of diet (HFC vs LFC) on faecal pellet production in the aversive OF test (t_(24)_ = 0.5527, *p* = 0.5856). In addition, *L*. *brevis* had no effect on faecal pellet production (F (2, 40) = 0.923, *p* = 0.406).

In contrast, diet and bodyweight did not impact mobility, represented by total distance travelled during the OF test conducted during the habituation phase (Day 1) of the NOR test. In this open arena, there was no difference in mobility between the HFC and LFC (Supplementary Fig. S1D). *L*. *brevis* had a significant impact on distance travelled during this test (F_2,38_ = 8.911, *p* < 0.001; Supplementary Fig. S1D) and it was revealed that DPC6108 increased total distance travelled, compared to the HFC (*p* < 0.001; Supplementary Fig. S1D). There was no effect of diet on duration of time spent in inner (Supplementary Fig. S1E) or outer (Supplementary Fig. S1F) zones of the NOR open field arena, between the HFC and LFC. Similarly, *L*. *brevis* had no effect on time spent in inner (Supplementary Fig. S1E) or outer (Supplementary Fig. S1F) zones of the NOR open field arena.

### *L. brevis* DSM32386 had a modest effect on cognitive function during the NOR test

During day two of the NOR test, all groups spent equal proportions of time investigating the two identical objects (A and B) within the open arena (Supplementary Fig. S2A). During day three, following introduction of a novel object in the space which was previously allocated to object B, while all mice increased the interaction time spent with the novel object, the effect was only significant following intervention with DSM32386 (t_(26)_ = 3.317, *p* < 0.01; Supplementary Fig. S2B). There was no impact of diet or *L*. *brevis* on the ability of mice to discriminate between the novel and familiar objects, as reflected in the discrimination index (F_(2,40)_ = 1.514, *p* = 0.233) (Supplementary Fig. S2C).

### GABA-producing microbes had no impact on anxiety-like behaviour during the elevated plus maze (EPM) test

In all aspects on the EPM test, there was no impact of diet or bodyweight identified with respect to the number of open (Supplementary Fig. S3A) (t_24_ = 1.353, *p* = 0.1887) and closed (Supplementary Fig. S3B) (t_(24)_ = 0.0647, *p* = 0.9468) arm entries for the HFC, compared with the LFC. Non-parametric analysis on the % of time spent in open (Mann-Whitney U value: 78.50; Wilcoxon W: 169.50 *p* = 0.758) and closed arms (t_24_ = 0.3962, *p* = 0.6955) did not reveal any impact of diet on anxiety-like behaviour (Supplementary Fig. S3C,D). Similarly, *L*. *brevis* had no effect on anxiety-like behaviour as measured by the % of time spent in open (Chi squared = 1.772; df = 2; p = 0.0412 [Kruskall-Wallis test]) and closed arms (F_2,40_ = 0.085, *p* = 0.918; Supplementary Fig. S3C,D).

## Discussion

This exploratory study demonstrates that daily administration of GABA-producing lactobacilli has a moderate efficacy in improving insulin sensitivity and glucose tolerance in metabolically dysfunctional mice. Two GABA-producing *L*. *brevis* strains of different origins were selected to compare their therapeutic potential in a mouse model of diet-induced obesity. The ability of certain lactic acid bacteria to biosynthesize GABA is a strain-dependent attribute, genetically governed by the genes *gadB* and *gadC*, whereby the GABA-production efficiencies differ between strains^34,35^. The GABA-production capability of *L*. *brevis* DSM32386 was comparable to that of *L*. *brevis* DPC6108 *in vitro*. However, DSM32386 appeared to have a greater impact, albeit modest, in improving some of the metabolic and behavioural outcomes associated with metabolic dysfunction in this mouse model.

GABA is synthesized from glutamate by glutamic acid decarboxylase (GAD) and is produced by pancreatic β-cells^36^. Once released, GABA binds the GABA_A_ receptor (ligand-gated Cl^−^ ion channel) on pancreatic α-cells, causing Cl^−^ influx following membrane hyperpolarization, in turn suppressing glucagon secretion^37,38^. It has been suggested in diabetic subjects that an impaired insulin-Akt-GABA_A_ receptor-glucagon secretory pathway in the islet may be an underlying mechanism for unsuppressed glucagon secretion, despite hyperglycaemia^38,39^. Unlike in α-cells, GABA_A_ receptor signalling in β-cells induces membrane depolarization, which opens voltage-dependent calcium channels, stimulating insulin secretion. Therefore, microbial intervention with GABA-producing strains has the potential to agonise GABA_A_ receptors in the pancreas, thereby preventing unsuppressed glucagon secretion and stimulating insulin secretion.

In this study, 12 weeks of HF feeding was sufficient to induce glucose intolerance and insulin insensitivity. The results indicate that microbial intervention, particularly with *L*. *brevis* DSM32386, improved glucose homeostasis by attenuating diet-induced obesity associated hyperglycaemia and insulin insensitivity. During the glucose tolerance test, intervention with both *L*. *brevis* strains significantly enhanced insulin secretion in response to glucose load. This could represent an increase in the sensitivity of the endocrine response to promote glucose clearance following GABA-producing *L*. *brevis* intervention.

Although there were no differences in the final bodyweight gain between the HF diet fed groups, *L*. *brevis* had a significant effect on the distribution of adiposity. Intervention with both DSM32386 and DPC6108 resulted in less MAT accumulation, compared with the HFC. This is important as the oxidative phosphorylation potential of MAT is known to be far greater than that of SAT in humans^40^. Moreover, MAT mass is known to correlate well with the development of insulin resistance and T2D, a characteristic which SAT does not possess^41^. Therefore, it appears that GABA-secreting microbial interventions altered fat distribution in a manner which contributes beneficially to metabolic fitness. Interestingly, both DSM32386 and DPC6108 also reduced percentage body fat mass, compared with the HFC. While these outcomes are intriguing, the mechanisms involved are not entirely clear, although certain probiotic bacteria have been shown to attenuate HF diet-associated weight gain and enhance insulin sensitivity in previous studies^42,43^. For example, Schneeberger *et al*. have demonstrated that *Akkermansia muciniphila* was negatively associated with HF feeding, correlating strongly with changes in the expression of lipid metabolism-related genes and inflammation markers in adipose tissue^43^. Modification of immune cells in MAT has also been associated with beneficial effects on diabetes^44^. While the underlying mechanisms through which *L*. *brevis* reduces MAT in the host remain unknown, there is evidence to suggest that the therapeutic effects could be mediated by re-distribution of fat in the host, but this remains to be explored. Equally as intriguing is the impact of *L*. *brevis* on host lipid metabolism, as both strains were associated with reductions in fasting circulating cholesterol levels and an overall improvement in post-prandial cholesterolaemia. The cholesterol-lowering effects of certain probiotic bacteria have previously been linked with microbial bile acid modification^45^ and exopolysaccharide production^46,47^. However, this exploratory study is the first to demonstrate a potential role of microbial GABA-production in addressing diet-induced hypercholesterolaemia.

We hypothesized that HF feeding would significantly affect the gut microbiota composition of the mice in this study. Taking a metataxanomic approach, we aimed to determine whether improvements in the metabolic profile following microbial intervention could be attributed to a beneficial impact on the gut microbiota. Several studies have characterised the gut microbiota composition of diet-induced obese mice using next generation sequencing methods^48–51^. Interestingly, there appears to be a characteristic shift in the relative abundances of the two most abundant phyla following obesity, whereby there is an increase in Firmicutes and a decrease in Bacteroidetes. Our data demonstrated that the HF diet did indeed induce alterations in the intestinal microbial composition, significantly increasing Firmicutes, with a stable abundance of Bacteroidetes in the HFC and in the groups exposed to *L*. *brevis* and HF diet. As a consequence, the ratio of Firmicutes to Bacteroidetes was increased following HF diet consumption; however, *L*. *brevis* did not mitigate this shift. Lower Firmicutes to Bacteroidetes ratios are associated with reduced blood glucose levels or increased glucose tolerance^48,52^. Indeed, our data reflected this by showing that the LFC mice had a lower ratio of Firmicutes to Bacteroidetes and concordant glucose tolerance when compared with the HFC. Conversely, despite the fact that the ratio of Firmicutes to Bacteroidetes was similar to the HFC, intervention with DSM32386 significantly increased glucose tolerance. Actinobacteria were decreased in all groups fed the HF diet irrespective of microbial intervention. Deferribacteres decreased in all groups fed the HF diet compared with the LFC and again, microbial intervention did not significantly reverse that change. A high inter-group variation was observed in terms of *Odoribacter* abundance, although DSM32386 intervention trended towards a reversal. Members of the *Odoribacter sp*. can cause health problems in humans and animals, but it has been reported that they may be beneficial in the prevention of hypertension^53,54^ and modulating host blood glucose levels^55,56^. However, *L*. *brevis* did not modulate the genus *Akkermansia* and thus does not explain the improved metabolic profile. Despite the low relative abundance of *Lactobacillus*, the OTUs belonging to this genus were manually blasted, and the species *L*. *brevis* was only found in the DSM32386 and DPC6108 groups.

A further objective was to identify whether changes in specific microbial genera were related to any metabolic parameters measured during the study. Considering both microbial and metabolic factors, the LFC and *L*. *brevis* groups clearly separated from the HFC on PCA. *Parabacteroides* appeared to be related to the increase of glycaemia after IP-ITT, the reduction of resting insulin levels and the increase of cholesterolaemia. *Bifidobacterium* and *Muribaculum* were related to reduced glycaemia (upon IP-GTT and/or IP-ITT) and the reduction of cholesterolaemia. Finally, *Erysipelotrichaceae* was related to reduced cholesterolaemia, and *Odoribacter* appeared to be related to reduced glycaemia (upon IP-GTT and/or IP-ITT). Since those genera tended to be increased in the LFC and *L*. *brevis* groups, respectively, they could be involved in the metabolic response of metabolically dysfunctional mice, although until proven, this point remains hypothetical. Nevertheless, if the gut microbiota changes would be the only correlation with the changes identified in the metabolic parameters, then intervention with DSM32386, which had improved metabolic parameters, would have had a different microbial profile than DPC6108. Thus, we hypothesize that the changes in the metabolic parameters observed in DSM32386 may be related to the higher luminal content of GABA in the small intestine and endogenous microbial GABA production. Therefore, alterations to the gut microbiota composition following *L*. *brevis* intervention do not solely explain the improvements to metabolic function.

The targeted analysis of >140 acylcarnitines, phosphatidylcholines, amino acids and biogenic amines revealed just two significantly altered metabolites. We observed an increase in ornithine for both microbial intervention groups and a decrease in arginine for DSM32386 alone. Both arginine and ornithine represent important amino acid metabolites in the arginine-proline metabolic pathway, which ultimately enters into the butanoate metabolic pathway through which GABA is synthesised in both *Mus musculus* and model bacterium *E*. *coli* K-12 MG1655. However, perhaps of greater relevance is the fact that ornithine is directly exchanged for arginine in lactobacilli in order to sustain the arginine deaminase pathway which contributes to GABA production^57^. In line with this, cultures of such bacteria will invariably produce ornithine and deplete arginine from their environment. Although this may indeed be an important contributor to the observed result, the implications for the host remain unclear.

Mounting literature describes the role of the gut microbiota and microbial intervention in the bidirectional communication system of the microbiome-gut-brain axis^29,58^. For example, intervention with specific bacterial strains has proven effective in modulating behaviour and brain function in animal models ranging from metabolic syndrome^59,60^ and depression^61^, to neurodevelopmental disorders such as autism^62^. Bacteria can produce a range of signalling molecules, or metabolites, including neuroactive amines and amino acids such as GABA, within the intestine which may signal to central and peripheral neural networks^30^. Obesity and stress-related psychiatric disorders are frequently comorbid. Clinical data from patients with metabolic syndrome and pre-clinical data from animal models of obesity have demonstrated an increased risk for neuropsychiatric disorders^25,26^. In addition, maternal obesity carries significant risks for offspring in terms of metabolic disturbances and behavioural deficits that manifest later in life^63,64^. Considering the associated risk of behavioural abnormalities associated with obesity, we aimed to identify whether microbial GABA production in the intestine could relieve any behavioural abnormalities associated with obesity and metabolic dysfunction. Previously, *L*. *rhamnosus* JB-1 induced region-dependent alterations in GABA_B1β_ and GABA_Aα2_ mRNA expression in a number of brain regions which correlated with an improvement in anxiety- and depression-related behaviour in mice^30^. Moreover, a recent clinical study investigating 16S microbiome sequencing and MRI brain scans of 23 patients suffering from major depressive disorder demonstrated a strong inverse correlation between relative abundances of a GABA-producing taxa, *Bacteroides*, and a key imaging feature of the disorder^65^. Neither HF diet nor *L*. *brevis* intervention had any impact on anxiety-related behaviour as measured by the OF and EPM tests. While mice in the DSM32386 group produced less faecal pellets during the FST, compared to the HFC, the method of counting faecal pellets produced during the FST is a particularly crude measure of anxiety-related behaviour and is not typically used as a robust output from this test which has been traditionally used to assess antidepressant-like activity preclinically^66^. Furthermore, there was no effect of *L*. *brevis* on faecal pellet production in the aversive OF test and we cannot conclude that either strain had any effect on anxiety-like behaviour. Intervention with DSM32386 had a modest effect towards improving recognition memory in the NOR test. This is particularly interesting since hyperglycaemia is a risk-factor for cognitive impairment^59,67,68^. One study previously demonstrated that impaired spatial memory in streptozotocin-induced diabetic rats was considerably improved following probiotic intervention^59^. Finally, *L*. *brevis* improved HF diet-induced behavioural despair as observed in the FST and basal corticosterone levels taken before the induction of FST-induced stress were lower following *L*. *brevis* intervention, compared with the HFC. Interestingly, the HF diet increased basal corticosterone in the HFC, compared to the LFC. This effect of HF feeding alone has been observed previously for both inducing despair behaviour and increasing corticosterone, compared to control diet^69^. The observation that both DPC6108 and DSM32386 reduced both despair behaviour and basal corticosterone levels compared to the HFC to levels which were comparable to the LFC is intriguing and opens the possibility that these strains could modulate brain behaviour and signalling, mediated through microbial GABA production.

## Conclusions

The data presented herein outline a potential role of metabolic- and neuroactive-microbial metabolite production in the modulation of diet-induced metabolic dysfunction, including abnormal behaviour. Supplementation with GABA-producing *L*. *brevis* increases endogenous GABA concentration in the small intestine with an impact on host health. Microbial modulation of the gut microbiota is a safe and effective means to modulate and improve health outcomes in this rodent model of obesity and metabolic dysfunction. This dataset describes that increased microbial GABA production can impact host metabolism and behaviour. Further substantiation of these exploratory health benefits in mice need to be confirmed before moving towards the clinic. This is the first study to our knowledge identifying a potential role of microbial GABA-production in alleviating some symptoms of metabolic dysfunction. Future experiments should utilise this dataset to focus on the potential mechanism of action of *L*. *brevis* DPC6108 and DSM32386 on the outcomes observed in this study. Furthermore, the potential for other metabolites to have a causative effect on the outcomes observed in this study should be explored.

## Materials and Methods

### Animals and treatment

All experimental procedures were performed in accordance with the protocols approved by the University College Cork Ethics Committee, under a license issued from the Health Products Regulatory Authority (AE19130/P026). Male C57BL/6J mice, three weeks of age, were obtained from Envigo (Blackthorn, UK) and housed under barrier-maintained conditions at the Biological Services Unit, UCC. All mice acclimatised to their environment for five weeks prior to administration of diets. Mice were randomly divided into four groups (*n* 14 per group) and housed in groups of three to four mice per cage at standard conditions (room temperature of 21 °C, with a 12-h light–dark cycle, lights on at 07:00).

The groups were assigned as follows;

#### Dietary intervention

A low fat (LF) group fed *ad libitum* with Open Source Diets (D15072701 – 10% kcal from fat; (Research Diets Inc., NJ 08901 USA)) and three high fat (HF) groups fed *ad libitum* with Open Source Diets (D12492 – 60% kcal from fat; Research Diets Inc.) were allowed free access to food and water, for 12 weeks. The compositions of the diets are outlined in Supplementary Table S1. After 12 weeks of either LF or HF feeding, two control groups were maintained on either the LF diet (LFC; *n* 14) or HF diet (HFC; *n* 14) for a further 12 weeks and the remaining two HF dietary groups were subdivided into intervention groups for a further 12 weeks.

#### Continued dietary intervention + microbial intervention

The HF intervention groups were as follows; (1) HF + *L*. *brevis* DPC6108 (1 × 10^10^ CFU/day; DPC6108) and (2) HF + *L*. *brevis* DSM32386 (1 × 10^10^ CFU/day; DSM32386). All interventions were administered daily in drinking water. Water containing either *L*. *brevis* DPC6108 or *L*. *brevis* DSM32386 was the only water supplied to the mice in these groups for the 12 week intervention period and bottles were replaced daily. Bodyweight and food intake were measured weekly for all groups. Following 8 weeks of intervention, all mice were subjected to the behavioural test battery to measure anxiety- and depressive-like behaviour and cognitive function. Mice were then allowed to recover for one week and after 10 weeks of intervention, all mice underwent the metabolic profiling battery. Following 12 weeks of intervention, mice were fasted overnight and body mass was measured using a Minispec mq benchtop NMR spectrometer (Bruker Instruments, Germany). The mice were subsequently sacrificed by cervical dislocation. All dissected tissue samples were flash frozen immediately in liquid nitrogen. Individual blood samples were collected in microtainer^TM^ collection tubes containing ethylenediaminetetraacetic acid (EDTA) (BD Microtainer Plasma Separator Tubes, BD Diagnostics, Oxford, UK), thoroughly mixed in the tube and stored on ice for 10 min at 2,000 *g* under refrigerated conditions to isolate the plasma. Isolated plasma was immediately transferred to a clean eppendorf tube following centrifugation.

### Preparation and administration of *L. brevis* DPC6108 and DSM32386

*L*. *brevis* DPC6108 and *L*. *brevis* DSM32386 are efficient GABA producers, with maximum conversion *in vitro* when grown on MRS broth supplemented with 3% (w/v) monosodium glutamate (MSG)^31^. Rifampicin-resistant variants of *L*. *brevis* DSM32386 and *L*. *brevis* DPC6108 were isolated by spread-plating ~10^9^ CFU from an overnight culture (1% inoculum) onto MRS agar (Difco Laboratories) containing 500 µg rifampicin/mL (Sigma-Aldrich Ireland Ltd. Arklow, Ireland) and stocked at −80 °C. Before freeze-drying, frozen stocks were plated on MRS agar and single colonies were isolated for inoculation in 10 mL fresh MRS broth supplemented with 30 mg/mL MSG. The cultures were incubated overnight at 37 °C under anaerobic conditions and then inoculated into 1 L MRS broth containing 3% (w/v) MSG and allowed to grow overnight at 37 °C, under anaerobic conditions. The overnight culture aliquots were then inoculated into large volumes of MRS containing MSG and allowed to grow overnight at 37 °C under anaerobic conditions. The overnight cultures were washed twice in phosphate buffered saline (Sigma-Aldrich Ireland Ltd.) and the pellets re-suspended in 15% (w/v) trehalose (Sigma-Aldrich Ireland Ltd.) in dH2O. One-millilitre aliquots of bacterial cultures were freeze-dried by using a 24-h program (freeze temperature, −40 °C; condenser set point, −60 °C; vacuum set point, 600 mTorr). Freeze-dried aliquots were prepared every two weeks, continuously underwent quality control checks and were stored at 4 °C until use. Vials of freeze-dried microbial powder were found to contain 2 × 10^10^ CFU following lyophilization. With an average calculated fluid intake of ~5 ml/mouse/day, a dose of ~1 × 10^9^ CFU/day was achieved by suspending a single vial in 100 ml of distilled water. This dilution delivered a concentration of 2 × 10^8^ CFU/ml. During re-suspension, 100 mL of sterile water containing the freeze-dried powder was vortexed extensively to ensure solubility and even dispersion of the powder in water. Fresh water containing the live microorganisms was administered to mice each day.

### Culture dependent microbial analysis

To confirm that *L*. *brevis* DPC6108 and *L*. *brevis* DSM32386 tolerated freeze-drying conditions, the strains were plated on MRS agar supplemented with rifampicin before and after freeze-drying. Fresh faecal samples were taken for microbial analysis to verify strain survival following gastric transit. A detailed description of the methods for culture dependent microbial analysis following gastric transit can be found in Supplementary Methods.

### Metabolic profiling battery

After 12 weeks of feeding, an intraperitoneal-glucose tolerance test (IP-GTT) and an intraperitoneal-insulin tolerance test (IP-ITT) was performed in the LFC (*n* 7) and HFC (*n* 7) groups.

Following confirmation of metabolic disturbance in the HFC group, the IP-GTT and IP-ITT were performed in individual mice from groups LFC, HFC, DPC6108 and DSM32386 after 10 weeks of intervention. The insulin resistance index was also calculated from the plasma insulin concentrations that were measured in plasma collected from tail blood during the IP-GTT and a mixed-meal tolerance test was performed, also after 10 weeks of intervention from individual mice in the LFC, HFC, DPC6108 and DSM32386 groups. A detailed description of the methods for the glucose and insulin tolerance tests, determination of insulin resistance index and the mixed meal tolerance test can be found in Supplementary Methods.

### GABA assay

Frozen samples of epididymal adipose tissue and small intestinal content were diluted (10%, (w/v)) in tissue lysis solution (0.01 N HCl, 1 mM EDTA, 4 mM sodium metabisulphite (Sigma-Aldrich Ireland Ltd.)) and homogenised. A commercially available enzyme immunoassay was then used for the quantitative determination of GABA by ELISA (ImmuSmol, Pessac, France). Quantification of unknown samples was achieved by comparing their absorbance with a standard curve prepared with known standards and results were standardised to individual sample weights.

### Plasma Metabolome – direct flow injection and LC-MS/MS

Plasma was isolated from whole blood, as previously described and analysed using the Biocrates AbsoluteIDQ p180 Kit (BIOCRATES Life Sciences AG, Austria), using methods previously described^70^. Following extraction and derivatisation, all analytes present in the samples were detected and quantified on an ABI 4000 Q-Trap mass spectrometer (MDS Sciex) run in conjunction with a reverse-phase HPLC-column. The analysis revealed levels of a range of specific amino acids, biogenic amines (BA), acylcarnitines (AC), lysophosphotidylcholines (lysoPC), phosphotidylcholines (PC), sphingomyelins (SM) and hexoses.

### Microbial DNA extraction, 16S rRNA amplification and Illumina Miseq sequencing

Caecal contents were collected from individual mice following 12 weeks of dietary intervention. Total metagenomic DNA was extracted from caecal contents with the QIamp® PowerFecal® DNA Kit (Qiagen, Milan, Italy) where an additional bead beating step was incorporated into the protocol. Extracted DNA was quantified using the NanoDropTM 8000 Spectrophotometer (Thermo Fisher Scientific). Total genomic DNA was then subjected to PCR amplification by targeting a 464-bp fragment of the 16S rRNA variable region V3-V4^71,72^ using the specific bacterial primer set 341F (5′-CCTACGGGNGGCWGCAG-3′) and 806R (5′-GACTACNVGGGTWTCTAATCC-3′) with overhang Illumina adapters. Unique barcodes were attached to the forward primer for facilitating the differentiation of samples. Amplicons were cleaned with the Agencourt AMPure kit (Beckman Coulter) following the manufacturer’s instructions, and DNA was quantified using the Quant-iT PicoGreen dsDNA kit (Invitrogen). Amplicons were mixed and combined in equimolar ratios, and the quality and purity of the library was checked with the High Sensitivity DNA Kit (Agilent, Palo Alto, CA, USA) by the Bioanalyzer 2100 (Agilent). The library was sequenced on an Illumina MiSeq platform at CIBIO (Center of Integrative Biology) – University of Trento, Italy. The bioinformatic analysis that was used to analyse the 16S rRNA Illumina Miseq sequencing data can be found in the Supplementary Methods.

### Behaviour test battery

Following 20 weeks of high fat feeding and eight weeks of *L*. *brevis* intervention, mice from all groups underwent a behavioural test battery. A detailed description of the methods for each behaviour test and stress-induced corticosterone production can be found in Supplementary Methods.

### Statistical analyses

All data are expressed as mean ± SEM. Data were analysed using the appropriate unpaired student t-test and one-way analysis of variance (ANOVA). Bodyweight data were analysed using the mixed model ANOVA and Tukey’s post-hoc test was applied. Data were deemed significant when *p* < 0.05. Levenes test for homogeneity of variances was used and where homogeneity was not found, Welch’s robust test of equality of means was applied. Mauchly’s test of sphericity was used and if values were significant, then Greenhouse Geisser was applied. All student t-tests, one-way ANOVA, and post hoc analyses were performed using PASW Statistics 22. The correlation matrix of microbiota profile data and metabolic parameters was performed using *Pearson*’*s Product-Moment Correlation*. Graphs were generated using Graphpad Prism 7. Metabolomic data was log normalised prior to ANOVA and multivariate analysis (sPLS-DA) was also performed on metabolomic data. For metabolomic data, a false discovery rate (FDR) q value less than 0.05 was considered statistically significant.

## Supplementary information

Supplementary information

## Data Availability

The data generated by Illumina sequencing were deposited in the NCBI Sequence Read Archive (SRA) and are available under Ac. No. PRJNA414526.
