# Supplementary material for: Gamma-aminobutyric acid-producing lactobacilli positively affect metabolism and depressive-like behaviour in a mouse model of metabolic syndrome
Source: Sci Rep. 2019 Nov 8;9:16323. doi: 10.1038/s41598-019-51781-x (PMC6841999; doi:10.1038/s41598-019-51781-x)
Supplement: Supplementary file 1 — Supplementary information [file 41598_2019_51781_MOESM1_ESM.pdf]

Gamma-aminobutyric acid-producing lactobacilli positively affect metabolism and depressive-like behaviour in a mouse model of metabolic syndrome

E. Patterson[1,2#], P. M. Ryan[1,2#], N. Wiley[1,2], I. Carafa[1,2,3], E. Sherwin[1], G. Moloney[1], E. Franciosi[3], R. Mandal[4], D. S. Wishart[4,5,6], K. Tuohy[3], P. Ross[1,7], J.F. Cryan[1,8], T.G. Dinan[1,9] and C. Stanton[1,2,*]

1 APC Microbiome Ireland, University College Cork

2 Teagasc Food Research Centre, Moorepark, Fermoy, Cork, Ireland

3 Department of Food Quality and Nutrition, Research and Innovation Centre-Fondazione Edmund Mach, San Michele all'Adige, Trento, Italy

4 Department of Biological Sciences, University of Alberta, Edmonton, Alberta, Canada

5 Department of Computing Science, University of Alberta, Edmonton, Alberta, Canada

6 National Institute for Nanotechnology, Edmonton, Alberta, Canada

7 College of Science, Engineering and Food Science, University College Cork, Cork, Ireland

8 Department of Anatomy and Neuroscience, University College Cork, Cork, Ireland

9 Department of Psychiatry and Neurobehavioural Science, University College Cork, Cork, Ireland

#Authors contributed equally to this work.

*Corresponding Author:

Prof Catherine Stanton

Email: Catherine.Stanton@teagasc.ie

Telephone: +353 (0)25 42606

Supplementary Methods

Culture dependent microbial analysis

Pooled fresh faecal samples collected from each cage of mice were analysed in duplicate following 2 and 5 weeks of intervention. Microbial analysis involved enumeration of *L. brevis* DSM32386 and *L. brevis* DPC6108 strains after plating serial dilutions on MRS agar supplemented with 100 µg rifampicin/mL (Sigma-Aldrich Ireland Ltd.) and incubating anaerobically for 48 h at 37°C. In addition, isolated colonies were tested for GABA production *in vitro*, as described previously[1]. Briefly, isolated colonies were grown anaerobically in MRS containing 3% (w/v) and 1% (w/v) MSG at 37°C for 55 h. Samples were then deproteinized by mixing equal volumes of 24% (w/v) trichloroacetic acid (TCA) and culture, allowed to stand for 10 min and centrifuged at 14,000*g* for 10 min. Supernatants were removed and diluted with 0.2 mol/L sodium citrate buffer, pH 2.2 to yield 250 nmol of each amino acid residue. Samples were then diluted with the internal standard, norleucine, to yield a final concentration of 125 nm/mL. Amino acids were quantified using a Jeol JLC-500/V amino acid analyser (Jeol Ltd, Garden City, Herts, UK) fitted with a Jeol Na^+ high-performance cation exchange column. To calculate the % bioconversion of 1% MSG to GABA the following calculation was used:

Glutamate in MRS (nmol/mL) – Glutamate in sample (nmol/mL) = nmol/mL of MSG consumed

(GABA in sample (nmol/mL) / nmol/mL of MSG consumed) *100

Glucose and insulin tolerance tests

After 12 weeks of feeding, an intraperitoneal-glucose tolerance test (IP-GTT) and an intraperitoneal-insulin tolerance test (IP-ITT) was performed in the LFC (*n* 7) and HFC (*n* 7) groups. After 10 weeks of intervention, the IP-GTT and IP-ITT were performed on individual mice in the LFC, HFC, DPC6108 and DSM32386 groups. For the IP-GTT, mice were injected with a glucose load (1g/Kg body weight) directly into the peritoneal cavity, following a 6 h fast. Blood glucose levels were measured before and 15, 30, 60, 90 and 120 min after glucose load. For the IP-ITT, mice were injected with an insulin load (0.75IU/g bodyweight) directly into the peritoneal cavity, following a 6 h fast. Blood glucose levels were measured before and 15, 30, 60, 90 and 120 min after insulin load. The concentration of blood glucose during the IP-GTT and IP-ITT was determined using a glucose meter (Accu-Chek Aviva, Roche Diabetes Care Ltd., West Sussex, UK) on blood samples collected from the tip of the tail vein.

Insulin resistance index

The plasma insulin concentrations were measured in plasma collected from tail blood during the IP-GTT, after 10 weeks of intervention, using a Mouse Insulin ELISA kit (Mercodia, Uppsala, Sweden), according to the manufacturer's instructions. The insulin resistance index was determined by multiplying the area under the curve of both the blood glucose (0 to 120 min) and the plasma insulin (0 to 15 min) obtained from the IP-GTT.

Mixed-meal tolerance test

A mixed-meal tolerance test was performed after 10 weeks of intervention. Mice were fasted for 6 h and a baseline blood sample was taken from the tail following tail incision and collected into EDTA tubes (BD Diagnostics). Mice were then administered 200µl of Ensure Plus liquid diet (1.5kcal/mL, 29.5% fat; Abbott Nutrition, Dublin, Ireland) by intragastric gavage. Blood was collected 2, 4 and 18 h post-gavage. Individual blood samples were collected in microtainer™ collection tubes containing ethylenediaminetetraacetic acid (EDTA) (BD Microtainer Plasma Separator Tubes, BD Diagnostics), thoroughly mixed in the tube and stored on ice until centrifugation for 10 min at 2,000*g* to isolate the plasma. Isolated plasma was immediately transferred to a clean eppindorf tube following centrifugation. The plasma was then analysed for cholesterol concentration at time points T0, T2, T4 and T18 h (EnzyChrom colorimetric assay; Cambridge Biosciences, UK).

Bioinformatic analysis by QIIME

Sequences obtained from Illumina sequencing were processed using Quantitative Insights Into Microbial Ecology (QIIME) software package version 1.9 [2]. The paired-end reads were associated to the corresponding sample through the unique barcode and joined. Reads were further processed with the inclusion of quality filtering based on a quality score of > 20 followed by subsequent removal of sequences below length threshold [2]. UCLUST was then used for clustering the reads left into operational taxonomic units (OTUs) at 97% identity [3]. PyNAST [4] was used to align OTUs with a minimum alignment of 150 bp and 80% of minimum identity, and taxonomy was assigned by using Ribosomal Database Project (RDP) classifier 2.0.1 [5]. QIIME was used to generate alpha (Chao1, observed OTUs) and beta diversities (Bray Curtis) distance matrices, and principal coordinate analysis (PCoA) plots were generated based on the beta diversity distance matrices. The data generated by Illumina sequencing were deposited in the NCBI Sequence Read Archive (SRA) and are available under Ac. No. PRJNA414526.

Behaviour test battery

For all behavioural tests, mice were habituated to the testing room by placing home-cages in the test room for at least 30 min prior to testing. The same mice were assessed across all behavioural tests. The behaviour tests were completed over two weeks. All apparatus were cleaned with 70% (v/v) ethanol between mice in each test. A researcher remained in the testing room during each behavioural measure. All outputs were measured by an experimenter blinded to the experimental groups.

Aversive open field test

Following eight weeks of dietary intervention, mice were tested in the open field (OF) for anxiety-related behaviour and locomotor activity. The apparatus was a grey plastic open arena without any bedding (40 cm × 30 cm × 25 cm, *L* × *W* × *H*). At the beginning of each trial, mice were placed in the centre of the brightly illuminated (1,000 lux) open field arena. Mice were allowed 10 min free exploration in the box. During this time, behaviour was recorded using a video camera and the number of faecal pellets in the arena were counted as an index of anxiety. At the end of each trial, mice were returned to their home cages with littermates. Total activity and time spent in inner zone were analysed using a tracking system from recorded material (Ethovision, Noldus, Wageningen, The Netherlands).

Novel object recognition

Following eight weeks of dietary intervention, the novel object recognition (NOR) test was used to evaluate cognition (memory and learning) and was conducted as previously described [6,7]. Day 1, the habituation phase, was performed as the OF test (as described above) where no objects were placed in the grey plastic open arena (40 cm × 30 cm × 25 cm, *L* × *W* × *H*) under low light conditions (60 lux). Day 2, 24 h following the habituation/open field test, mice were reintroduced to the arena containing two identical objects placed in adjacent corners of the arena, approximately 5 cm from each wall. Mice were again allowed 10 min free exploration in the arena, during such time, behaviour was recorded using a video camera as above. Day 3, 24 h after day 2, mice were once again reintroduced to the arena, this time containing one familiar and one novel object, and again, mice were allowed free exploration of the arena for 10 min and during this time, behaviour was recorded using a video camera. After each phase mice were returned to their home cages with littermates.

The arena and objects were cleaned with 70% (v/v) ethanol between trials. Object exploration was defined as the time when the animal's nose comes within a 2-cm radius of the object. Memory was defined by the discrimination index for the novel object (DI) as the difference of time mice spent investigating between the novel and the familiar object divided by the total time exploring both objects. [Discrimination Index, DI = (Novel Object Exploration time - Familiar Object Exploration time) / (Novel Object Exploration time + Familiar Object Exploration time)].

Elevated plus maze

Following nine weeks of dietary intervention, mice were tested in the elevated plus maze (EPM) test to assess anxiety-like behaviour. The apparatus used was an elevated (1 m from the floor) cross plastic maze, comprising two closed 'safe' arms and two open 'fearful' arms (50 cm × 5 cm × 15 cm walls). Mice were individually placed into the centre of the maze facing an open arm to avoid direct entrance into a closed arm and left to explore for five minutes. Both the time spent in each arm, as well as the number of entries was scored manually (entrance in one arm being defined as all four paws inside the arm). At the end of each trial, mice were returned to their home cages with littermates.

Forced swim test

Following nine weeks of dietary intervention, depressive-like behaviour and stress responsiveness were assessed using the forced swim test (FST), as previously described [8]. Mice were individually placed in a transparent plexi-glass cylinder (24 cm x 21 cm, $H \times D$), containing 15 cm-depth water maintained at room temperature (22 ± 1°C) for a single six minute trial. Water was renewed between each trial. The total time of immobility was scored in the last four minutes [9]. Immobility was defined as the total absence of movement, except slight motions to maintain the head above water. After the trial, mice were gently dried and single-housed for two hours of recovery, before being placed back to their home cages with littermates.

Stress-induced corticosterone production

To assess stress-responsiveness, blood samples were taken in response to an acute stress (FST). First, a blood sample was collected from the tail following tail incision, five minutes before the test. After the acute stress, mice were singly housed following removal from the FST, and blood samples were collected at 15, 45, 90 and 120 minutes after the test.

Bleeding was performed in a separate room to the FST. Blood samples (50-70μl) were taken from the tail and collected in heparin coated capillary tubes. The blood was then transferred to a microtainer™ collection tubes containing EDTA (BD Diagnostics), thoroughly mixed in the tube and stored on ice until centrifugation for 10 min at 2,000g to isolate the plasma. Isolated plasma was immediately transferred to a clean eppindorf tube following centrifugation. Isolated plasma was stored at -80 °C for later corticosterone quantification. Corticosterone was quantified using a commercially available ELISA kit (Enzo Life Sciences (UK) Ltd., Exeter, UK) according to the manufacturer's protocol.

1 Barrett, E., Ross, R., O'toole, P., Fitzgerald, G. & Stanton, C. γ-Aminobutyric acid production by culturable bacteria from the human intestine. *Journal of applied microbiology* **113**, 411-417 (2012).

2 Caporaso, J. G. *et al.* QIIME allows analysis of high-throughput community sequencing data. *Nature methods* **7**, 335 (2010).

3 Edgar, R. C. Search and clustering orders of magnitude faster than BLAST. *Bioinformatics* **26**, 2460-2461 (2010).

4 Caporaso, J. G. *et al.* PyNAST: a flexible tool for aligning sequences to a template alignment. *Bioinformatics* **26**, 266-267 (2009).

5 Maidak, B. L. *et al.* The RDP-II (Ribosomal Database Project). *Nucleic Acids Res* **29**, 173-174 (2001).

6 Burokas, A., Gutiérrez-Cuesta, J., Martín-García, E. & Maldonado, R. Operant model of frustrated expected reward in mice. *Addiction biology* **17**, 770-782 (2012).

7 Pusceddu, M. M. *et al.* N-3 Polyunsaturated Fatty Acids (PUFAs) Reverse the Impact of Early-Life Stress on the Gut Microbiota. *PloS one* **10**, e0139721, doi:10.1371/journal.pone.0139721 (2015).

8 Cryan, J. F. *et al.* Use of dopamine-β-hydroxylase-deficient mice to determine the role of norepinephrine in the mechanism of action of antidepressant drugs. *Journal of Pharmacology and Experimental Therapeutics* **298**, 651-657 (2001).

9 Cryan, J. F., Markou, A. & Lucki, I. Assessing antidepressant activity in rodents: recent developments and future needs. *Trends in pharmacological sciences* **23**, 238-245 (2002).

Supplementary Tables and Figures:

Supplementary Table S1: Diet information

**Product #**	**D15072701**			**D12492**	
	gm	*Kcal (%)*		gm	*Kcal (%)*
Protein	19	*20*		26	*20*
Carbohydrate	67	*70*		26	*20*
Fat	4	*10*		35	*60*
Total		*100*			*100*
kcal/gm	3·8			5·2	
**Ingredient**	**gm**	***kcal***		**gm**	***kcal***
Casein	200	*800*		200	*800*
L-Cysteine	3	*12*		3	*12*
Corn Starch	280	*1120*		0	*0*
Maltodextrin 10	140	*560*		125	*500*
Sucrose	280	*1120*		68.8	*275*
Cellulose, BW200	50	*0*		50	*0*
Soybean Oil	25	*225*		25	*225*
Lard	20	*180*		245	*2205*
Mineral Mix S10026	10	*0*		10	*0*
DiCalcium Phosphate	13	*0*		13	*0*
Calcium Carbonate	5·5	*0*		5·5	*0*
Potassium Citrate, 1 H20	16·5	*0*		16·5	*0*
Vitamin Mix V10001	10	*40*		10	*40*
Choline Bitartrate	2	*0*		2	*0*
FD&C Yellow Dye #5	0	*0*		0	*0*
FD&C Red Dye #40	0·025	*0*		0	*0*
FD&C Blue Dye #1	0·025	*0*		0·05	*0*
**Total**	**1055·05**	***4057***		**773·85**	***4057***

Supplementary Table S2: Alpha and Beta Diversity Indexes

	Chao_1	OTUs (n)	Bray-Curtis
**LFC**	4397 ± 621[a]	2501 ± 383[a]	0.36 ± 0.06[a]
**HFC**	3599 ± 576[b]	1943 ± 330[b]	0.41 ± 0.06[b]
**DPC6108**	4138 ± 720[a]	2238 ± 494[a,b]	0.42 ± 0.09[b,d]
**DSM32386**	3375 ± 487[b]	1857 ± 328[b]	0.44 ± 0.08[c,d]

Supplementary Table S3: Diet appeared to be the main determinant of microbiota-biochemistry associations

	Glycaemia_GTT	Glycaemia_ITT	Insulin	Cholesterolemia
Bifidobacteria	↓	↓	-	↓
Parabacteroides	-	↑	↓	↑
Muribaculum	↓	↓	-	↓
Odoribacter	↓	↓	-	-
Bacteroidetes_other	-	-	-	↑
Erysipelotrichaceae	↓	-	-	↓

Supplementary Figure S1: *L. brevis* had no effect on anxiety-like behaviour in the aversive and NOR open field test

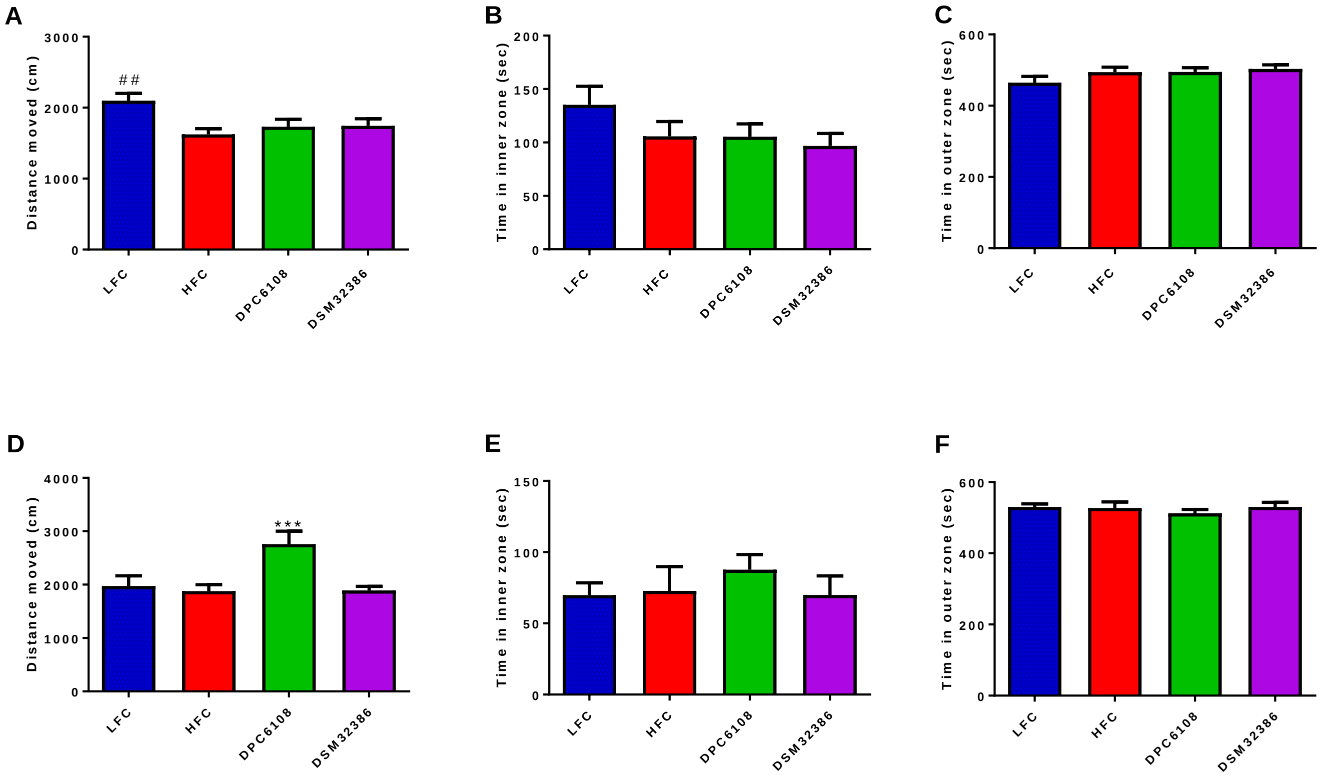

Supplementary Figure S2: *L. brevis* DSM32386 had a modest effect on object recognition behaviour / cognitive function in the NOR test

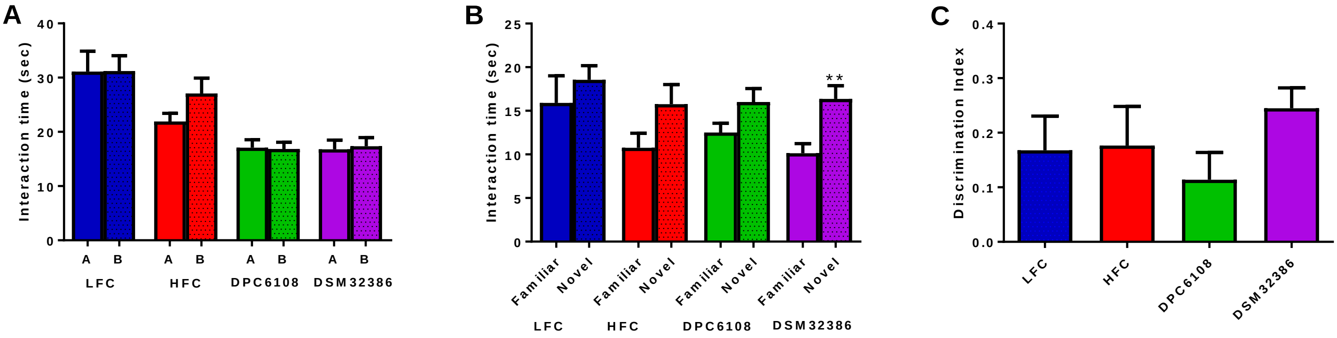

Supplementary Figure S3: *L. brevis* had no effect on anxiety-like behaviour in the EPM test

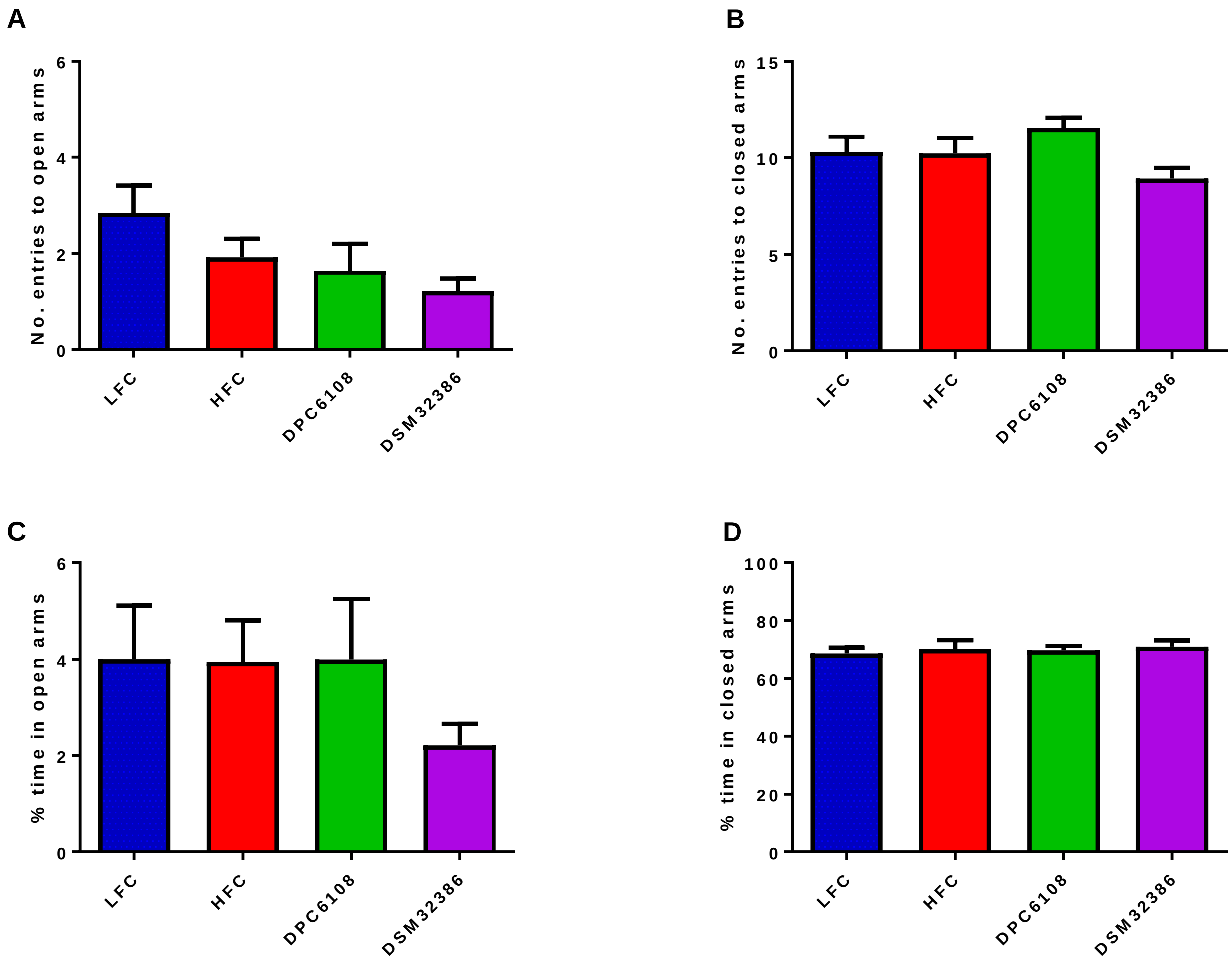

Supplementary Figure Legends

Supplementary Table S1: Mice were fed *ad libitum* with either a low fat diet (Open Source Diets (D15072701 – 10% kcal from fat and equal parts corn starch and sucrose; (Research Diets Inc., NJ 08901 USA)) or a high fat diet (Open Source Diets (D12492 – 60% kcal from fat; Research Diets Inc.)) and were allowed free access to food and water, for 24 weeks.

Supplementary Table S2: Alpha (Chao-1, numbers of observed OTUs) and Beta (Bray-Curtis) diversity indexes are shown as mean values ± standard deviation. Different letters indicate a significant difference (p <0.05).

Supplementary Table S3: Diet appeared to be the main determinant of microbiota-biochemistry associations. Correlation between the faecal microbiota and glycaemia during IP-GTT, glycaemia during IP-ITT, insulin or cholesterol levels in the blood. Green arrows indicate a negative correlation, while red arrows indicate a positive correlation (p <0.05). The dash indicates no significant correlation (p >0.05).

Supplementary Figure S1: *L. brevis* had no effect on anxiety-like behaviour in the aversive and NOR open field test. The effect of HF-diet feeding and probiotic interventions on anxiety-like behaviour was assessed after 21 weeks of feeding and after 8 weeks of intervention. Total distance moved **(A)**, time spent in the inner zone **(B)** and time spent in the outer zone **(C)** was measured in the aversive OF test and the same outcomes were again measured in the NOR OF test **(D, E and F, respectively)** for LFC (n=13), HFC (n=13), DPC6108 (n=14), DSM32386 (n=14). Data are expressed as mean ± SEM. All data was analysed using the appropriate unpaired student t-test (HFC vs LFC) and one-way analysis of variance (ANOVA). ## p < 0.01 HFC vs LFC, * p < 0.05 treatment vs HFC. HF: high fat, HFC: high fat control, DPC6108: *L. brevis* DPC6108, DSM32386: *L. brevis* DSM32386, LFC: low fat control.

Supplementary Figure S2: *L. brevis* DSM32386 had a modest effect on object recognition behaviour / cognitive function in the NOR test. The effect of HF diet feeding and microbial interventions on cognitive function was assessed after 21 weeks of feeding and 8 weeks of intervention. On day 1 of the test, mice were allowed to familiarise themselves with two identical objects **(A)**. On day 2, one of the familiar objects was replaced by a novel object **(B)** and the discrimination index represents how the mice could identify the change between the familiar and novel object during day 2 **(C).** The NOR test was performed on LFC (n=13), HFC

(n=13), DPC6108 (n=14), DSM32386 (n=14). Data are expressed as mean ± SEM. All data was analysed using the appropriate unpaired student t-test (HFC vs LFC) and one-way analysis of variance (ANOVA). ** $p < 0.01$ novel vs familiar. NOR: novel object recognition, HF: high fat, HFC: high fat control, DPC6108: *L. brevis* DPC6108, DSM32386: *L. brevis* DSM32386, LFC: low fat control.

Supplementary Figure S3: *L. brevis* had no effect on anxiety-like behaviour in the EPM test. The effect of HF-diet feeding and microbial interventions on anxiety-like behaviour was assessed after 21 weeks of feeding and after 8 weeks of intervention. The number of entries to the open **(A)** and closed **(B)** arms of the maze was assessed and the percentage of time spent in the open **(C)** and closed **(D)** arms was also calculated for LFC (n=13), HFC (n=13), DPC6108 (n=14), DSM32386 (n=14). Data are expressed as mean ± SEM. All data was analysed using the appropriate unpaired student t-test (HFC vs LFC) and one-way analysis of variance (ANOVA). EPM: elevated plus maze, HF: high fat, HFC: high fat control, DPC6108: *L. brevis* DPC6108, DSM32386: *L. brevis* DSM32386, LFC: low fat control.
